# mTORC1-mediated inhibition of polycystin-1 expression drives renal cyst formation in tuberous sclerosis complex

**DOI:** 10.1038/ncomms10786

**Published:** 2016-03-02

**Authors:** Monika Pema, Luca Drusian, Marco Chiaravalli, Maddalena Castelli, Qin Yao, Sara Ricciardi, Stefan Somlo, Feng Qian, Stefano Biffo, Alessandra Boletta

**Affiliations:** 1 Division of Genetics and Cell Biology, Dibit San Raffaele Scientific Institute, Via Olgettina, 58, Milano 20132, Italy; 2 PhD Program in Biology and Biotherapy of Cancer, Università Vita-Salute San Raffaele, Via Olgettina, 58, Milano 20132, Italy; 3 Division of Nephrology, Department of Medicine, University of Maryland School of Medicine, Baltimore, Maryland 21201, USA; 4 INGM, Via Sforza 28, Milano 20122, Italy; 5 Department of Internal Medicine and Department of Genetics, Yale University School of Medicine, New Haven, Connecticut 06520-8029, USA; 6 Department of Biosciences, University of Milan, Via Celoria, 26, Milano 20133, Italy

## Abstract

Previous studies report a cross-talk between the polycystic kidney disease (PKD) and tuberous sclerosis complex (TSC) genes. mTOR signalling is upregulated in PKD and rapamycin slows cyst expansion, whereas renal inactivation of the *Tsc* genes causes cysts. Here we identify a new interplay between the PKD and TSC genes, with important implications for the pathophysiology of both diseases. Kidney-specific inactivation of either *Pkd1* or *Tsc1* using an identical Cre (*KspCre*) results in aggressive or very mild PKD, respectively. Unexpectedly, we find that mTORC1 negatively regulates the biogenesis of polycystin-1 (PC-1) and trafficking of the PC-1/2 complex to cilia. Genetic interaction studies reveal an important role for PC-1 downregulation by mTORC1 in the cystogenesis of *Tsc1* mutants. Our data potentially explain the severe renal manifestations of the TSC/PKD contiguous gene syndrome and open new perspectives for the use of mTOR inhibitors in autosomal dominant PKD caused by hypomorphic or missense *PKD1* mutations.

## Abstract (web summary)

Polycystic kidney disease (PKD) is a ciliopathy resulting from defective localization of membrane proteins such as PC-1 to the primary cilium, resulting in renal cysts, and is associated with another cystic genetic disease called tuberous sclerosis complex (TSC). Here the authors use kidney-specific Tsc1 and Pkd1 mice to show that mTORC1 signalling inhibits PC-1 biogenesis as a potential mechanism of TSC/PKD contiguous gene syndrome.

## Graphical abstract

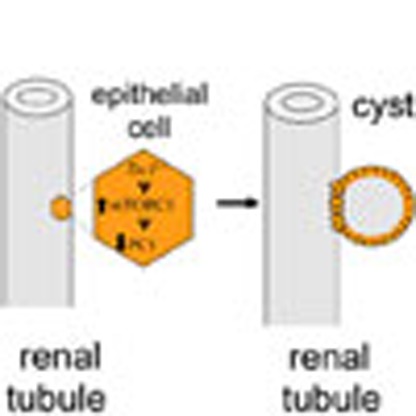

Autosomal dominant polycystic kidney disease (ADPKD) is a common genetic disorder characterized by massive bilateral renal cyst formation^1^. Two genes are associated with the disease: *PKD1* mutated in 85% of cases and *PKD2* mutated in the remaining 15% (refs 1, 2). ADPKD is a chronic condition characterized by the continuous formation and expansion of cysts arising from the epithelia lining the tubules of a minority of nephrons, which gradually causes compression and loss of function of all nephrons within a kidney. End stage kidney disease requiring renal replacement therapies ensue in 50% of affected individuals before age 60 (ref. 1). Intense studies in the past decade have lead to the identification of numerous signalling pathways that appear to be de-regulated in the cystic epithelia^1^,^2^. Several of these pathways and cascades have been considered potential good targets for therapy, irrespective of whether or not their defective regulation causes cyst formation or is caused by cyst formation^3^. Pathways that have been proposed to be de-regulated in PKD include Ca^++^ homoeostasis, cAMP upregulation, MAPK, mTOR and STAT signalling, sirtuins and TNF^1^,^2^. Prominent defective metabolic rates have also been described in ADPKD animal models, providing additional opportunities for therapy^3^,^4^. Although these studies have identified potential new targets for therapies, only one class (vasopressin receptor 2 antagonists) has reached the stage of approval for therapy in Japan, Canada and Europe^5^. Despite this progress, the primary cause of cyst formation remains elusive^3^.

Dysregulation of the mTOR pathway in ADPKD has attracted a great deal of attention both for the potential of using its inhibitors (rapalogues) as potential therapies and for the unusually intriguing cross-talk bewteen two genes mutated in different genetic disorders^6^,^7^,^8^,^9^,^10^. Several studies have implicated crosstalk between the *PKD* genes and the genes mutated in a genetic disorder called tuberous sclerosis complex (TSC)^6^,^7^,^9^,^10^. First, TSC patients can manifest with a variable degree of renal cysts^11^. Second, TSC is caused by mutations in either the *TSC1* or the *TSC2* genes and the proteins they encode are central regulators of the mTOR pathway^12^,^13^, which is hyperactive in some PKD mouse models and in some human cysts. Furthermore, the *PKD1* gene product polycystin-1 (PC-1), inhibits the mTORC1 cascade^8^,^9^,^14^. Treatment with rapamycin proved effective in retarding cyst growth in animal models of PKD^8^,^10^,^15^, although subsequent human clinical trials generated mostly negative results^16^,^17^,^18^. The possibility of cross-talk between PKD and TSC was first hypothesized on the basis of genetic evidence. The *PKD1* and *TSC2* genes are positioned tail-to-tail on the same chromosome, and large deletions causing disruption of both genes frequently result in massive and precocious renal cystic phenotypes in infants^19^. No mechanistic explanation has been proposed for this phenotype but previous studies showed that conditional inactivation of the *Tsc* genes in the mouse kidney results in renal cystogenesis^20^,^21^,^22^,^23^.

In response to these studies, some investigators have hypothesized that the mTOR pathway might play a more proximal role in cyst formation because of the similarities in the phenotype when the *Pkd* and the *Tsc* genes are inactivated in the kidney^21^,^22^. However, a direct comparison between the phenotype generated by inactivation of these two classes of genes by using the same Cre line has not been reported. Here, we show that inactivation of the *Tsc1* gene using a kidney-specific Cre line (Ksp:Cre) results in a much milder phenotype than inactivation of the *Pkd1* gene using the same Cre line. These data *per se* might suggest that mTOR is only one of the several pathways de-regulated by inactivation of the *Pkd1* gene and therefore the phenotype is not entirely recapitulated. In search for additional explanations for this difference in the phenotype, we unexpectedly found that the mTORC1 cascade regulates the expression of PC-1. Importantly, using genetic interaction studies we found that re-expression of *Pkd1* in the *Tsc1*-mutant kidneys corrects the phenotype. Our data show that TSC-PKD cross-talk is more complex than expected and that downregulation of the *PKD1* gene product might play an important role in cyst formation in TSC.

## Results

### Different time of mTORC1 upregulation and renal cystogenesis

**Figure 1:**
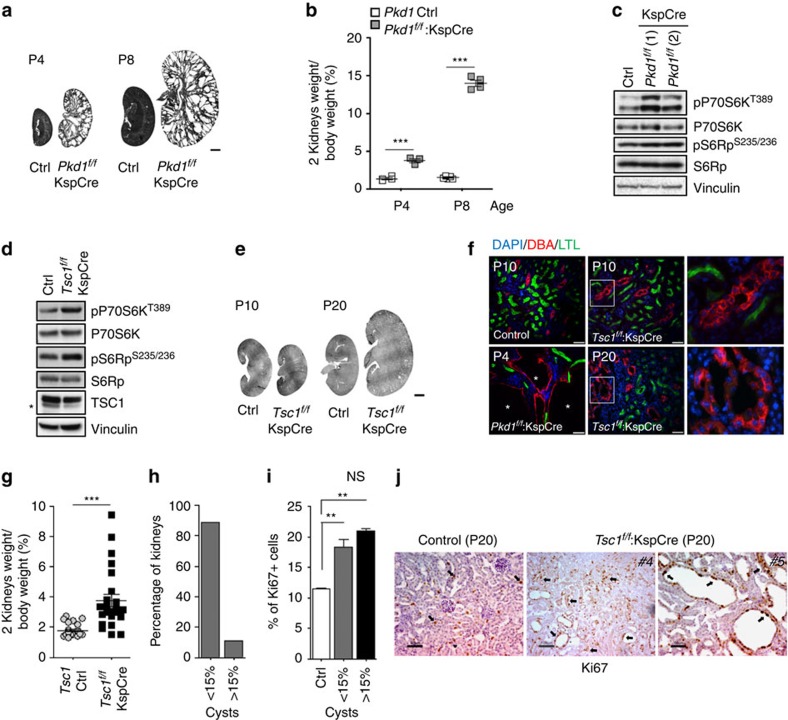
Inactivation of *Pkd1* and *Tsc1* leads to a different phenotype. (**a**) H&E staining of kidney sections from controls mice and *Pkd1*^*f/f*^*:KspCre* at P4 and P8. Scale bar, 1,000 μm. (**b**) Graph shows the two kidneys weight over body weight ratio for controls and *Pkd1*^*f/f*^:*KspCre* mice analysed at P4 (controls *n*=4, cystic *n*=3) and P8 (controls *n*=5, cystic *n*=4). Data are shown as mean±s.e.m. ANOVA statistical analysis (***P<0.0001) followed by Bonferroni's multiple comparison test was performed. ***P<0.001. (**c**) Representative western blot analysis of total kidney lysates from *Pkd1*^*f/f*^*:KspCre* mice compared to control shows upregulation of the mTORC1 cascade at P4. (**d**) Western blot analysis of total lysates from P10 *Tsc1*^*f/f*^*:KspCre* and control kidneys. Data are reperesentative of four independent experiments. (**e**) Representative H&E staining of kidney sections from *Tsc1*^*f/f*^*:KspCre* mice and controls at P10 and P20. Scale bar, 1,000 μm. (**f**) Immunofluorescence on kidney sections from *Pkd1* and *Tsc1* mutants performed with markers of the proximal (LTL, green) and distal tubules/collecting ducts (DBA, red). Scale bar, 45 μm. (**g**) Graph shows the two kidneys weight over body weight ratio in *Tsc1*^*f/f*^*:KspCre* mice (*n*=25) versus controls (*n*=26). Data are shown as mean±s.e.m. Student's unpaired two-tailed *t*-test was performed. ***P<0.001. F test was performed to compare variances. (**h**) Graph shows the distribution of P20 *Tsc1*^*f/f*^*:KspCre* (*n*=9) mice according to the percentage of cysts affecting the kidneys. (**i**) Graph shows the percentage of Ki67 positive cells in controls (*n*=3) and in *Tsc1*^*f/f*^*:KspCre* mice (cysts <15% *n*=6, cysts >15% *n*=3) performed at P20. A minimum of 5,000 cells per sample was counted (1,500 cells per section in three different sections in each sample). Data are shown as mean±s.e.m. ANOVA (***P=0.0007) followed by Dunnett's multiple comparison test was performed. **P<0.01, ***P<0.001. (**j**) Figure shows immunohistochemistry for Ki67 proliferation marker performed on kidney sections from control and *Tsc1*^*f/f*^:*KspCre* mice at P20. Arrows evidence Ki67 positive nuclei. Scale bar, 50 and 100 μm. ANOVA, analysis of variance.

To better understand cross-talk between the *TSC* and *PKD* genes in renal cyst formation, we intercrossed mice harbouring either *Pkd1* (ref. 24) or *Tsc1* (ref. 25) floxable alleles with an identical Cre line (*KspCre*)^26^, which is active in the distal tubules and collecting ducts starting at E14.5, both on the same genetic background (C57Bl/6). *Pkd1*^*flox/flox*^*:KspCre* mice manifest a fast and aggressive renal cystic phenotype causing death of the animals by P12 as previously reported (Fig. 1a,b)^4^,^27^. Biochemical analysis revealed that phosphorylation of S6K, a target of mTORC1, is increased in these kidneys at P4 when cysts are already visible (Fig. 1c). By contrast, *Tsc1*^*flox/flox*^*:KspCre* mice are born with normal kidneys. At P10, *Tsc1*^*flox/flox*^*:KspCre* mice show increased phosphorylation of S6K, showing upregulation of mTORC1 biochemically as expected (Fig. 1d and Supplementary Fig. 1a,b), but no evidence of renal cysts, except for a few tubular dilatations (Fig. 1e,f). Similar results were generated when comparing *Pkd1*^*flox/*-^*:KspCre* with *Tsc1*^*flox/*-^:*KspCre* mice (Supplementary Fig. 1c-f). Notably, at P20 *Tsc1*^*flox/flox*^*:KspCre* mice have an enhanced kidney/body weight, which is present in a high proportion of samples analysed (Fig. 1g), while cyst formation can be observed in only ∼20% of the animals (Fig. 1h). As previously reported, the appearance of the epithelia lining the cysts is morphologically different in *Pkd1* versus *Tsc1* mutant kidneys, with the first being characterized by a flat epithelium and the second by a columnar epithelium (Supplementary Fig. 1g). Analysis of proliferation using Ki67 as a marker for all cell-cycle phases revealed that upregulation of proliferation can be observed both in the cystic and in the non-cystic samples (<15%, Fig. 1i,j). These data indicate that increased proliferation alone does not seem to be sufficient to drive cystogenesis, similarly to what recently proposed^28^, although it is sufficient to cause kidney enlargement. Importantly, these data taken together show that there is a time-disconnect between mTORC1 upregulation and cyst formation. Thus, mTORC1 upregulation is not *per se* sufficient to recapitulate renal cystogenesis in *Pkd1* mutants, in line with the raising idea that alterations in a single pathway alone are not sufficient to recapitulate the cystic phenotype.

### mTORC1 downregulates PC-1 expression levels

**Figure 2:**
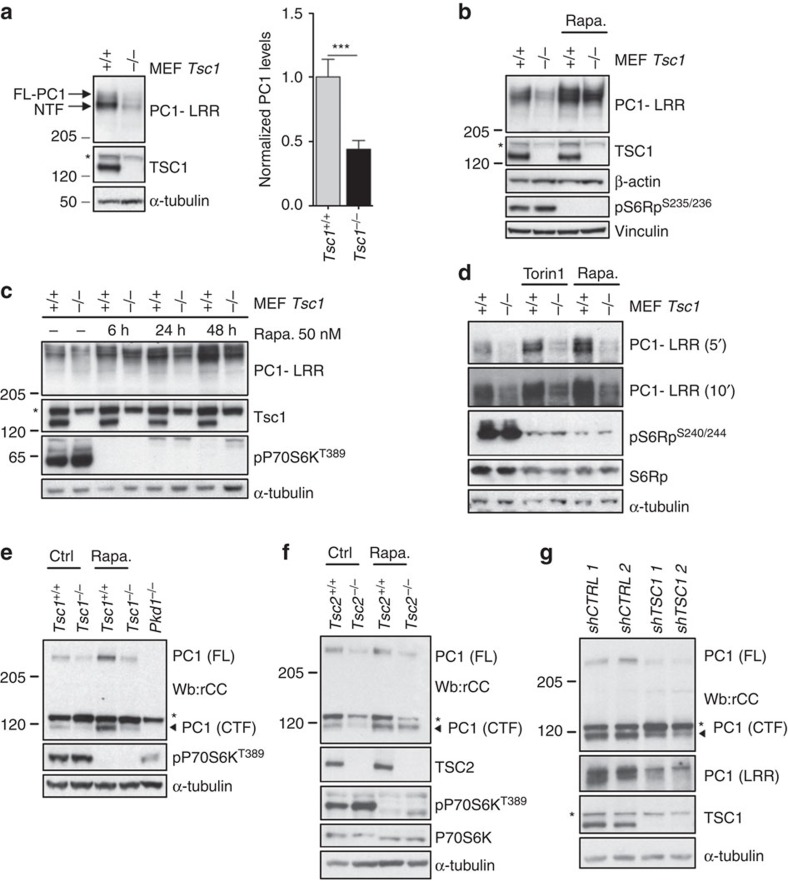
mTORC1 regulates polycystin-1 expression levels. (**a**) *Tsc1*^*+/+*^ and *Tsc1*^*−/−*^fibroblasts were immunoblotted for endogenous PC-1. Graph shows quantification of 12 independent experiments. Data are shown as mean±s.e.m. Statistical analysis was performed using the Student's unpaired two-tailed *t*-test. ***P=0.0009. F-test was used to compare variances. *P=0.0423. (**b**) Western blot analysis of PC-1 levels in *Tsc1*^*+/+*^ and *Tsc1*^*−/−*^cells cultured with or without 100 nM rapamycin. Data are representative of five independent experiments. *shows a non-specific band. (**c**) *Tsc1*^*+/+*^ and *Tsc1*^*−/−*^fibroblasts treated with or without 50 nM rapamycin for 6, 24 and 48 h and immunoblot-detecting PC1. Data are representative of two independent experiments. *shows a non-specific band. (**d**) *Tsc1*^*+/+*^ and *Tsc1*^*−/−*^ cells treated with Torin1 300 nM followed by immunoblot of PC1. (**e**) Immunoblot using an anti-C-terminal tail of PC-1 antibody (rCC)^30^ in *Tsc1*^*+/+*^ and *Tsc1*^*−/−*^ cells before and after 100 nM rapamycin. Arrows indicate the specific bands, * shows a non-specific band. (**f**) Immunoblot using an anti-C-terminal tail of PC-1 antibody (rCC)^30^ in *Tsc2*^*+/+*^ and *Tsc2*^*−/−*^ cells before and after 100 nM rapamycin. Arrows indicate the specific bands, * shows a non-specific band. (**g**) Immunoblot using an anti-C-terminal tail of PC-1 antibody (rCC)^30^ or against the LRR repeats (7e12) in two distinct clones of mIMCD3 cells infected with a shRNA directed against the *Tsc1* gene (shTSC1-1 and -2) or control shRNA sequences (shCTRL-1 and -2), * shows a non-specific band.

We next aimed at studying the *TSC/PKD* cross-talk using mouse embryonic fibroblasts (MEFs) derived from *Tsc1*^*−/−*^ embryos^29^. Using an antibody directed against PC-1, we unexpectedly observed a downregulation of PC-1 expression levels in *Tsc1* mutant as compared with control WT cells (Fig. 2a). Rapamycin restored the expression levels of PC-1 (Fig. 2b,c) in a time and dose-dependent manner (Fig. 2c, and Supplementary Fig. 2a,b), suggesting that mTORC1 can regulate PC-1 expression levels. *Pkd1* wild-type mouse embryonic fibroblasts derived from different litters in different labs, all responded to rapamycin treatment by upregulating PC-1 expression levels (Supplementary Fig. 2a,b). Furthermore, a different inhibitor of mTOR, Torin1 (300 nM), also restored PC-1 expression levels in *Tsc1* mutant cells and enhanced its expression in wild-type cells (Fig. 2d). A second antibody directed against the C-terminal tail of PC-1 (rCC)^30^ further confirmed the downregulation of PC-1 in *Tsc1*^*−/−*^ cells, but also in *Tsc2* mutant cell lines and in both cases rapamycin was able to restore normal expression levels (Fig. 2e,f). Finally, we generated a set of murine inner medullary collecting duct (mIMCD) cells carrying stable expression of shRNAs directed against the *Tsc1* gene. We achieved a 80% silencing level leading to upregulation of mTORC1 as expected in two stable transfectants as compared to scrambled transfected clones (Supplementary Fig. 2d). In these cell lines, as well, we observed a marked downregulation of PC-1 in cells carrying *Tsc1* silencing as compared with scrambled controls (Fig. 2g).

### mTORC1 regulation of PC-1 requires protein neo-synthesis

**Figure 3:**
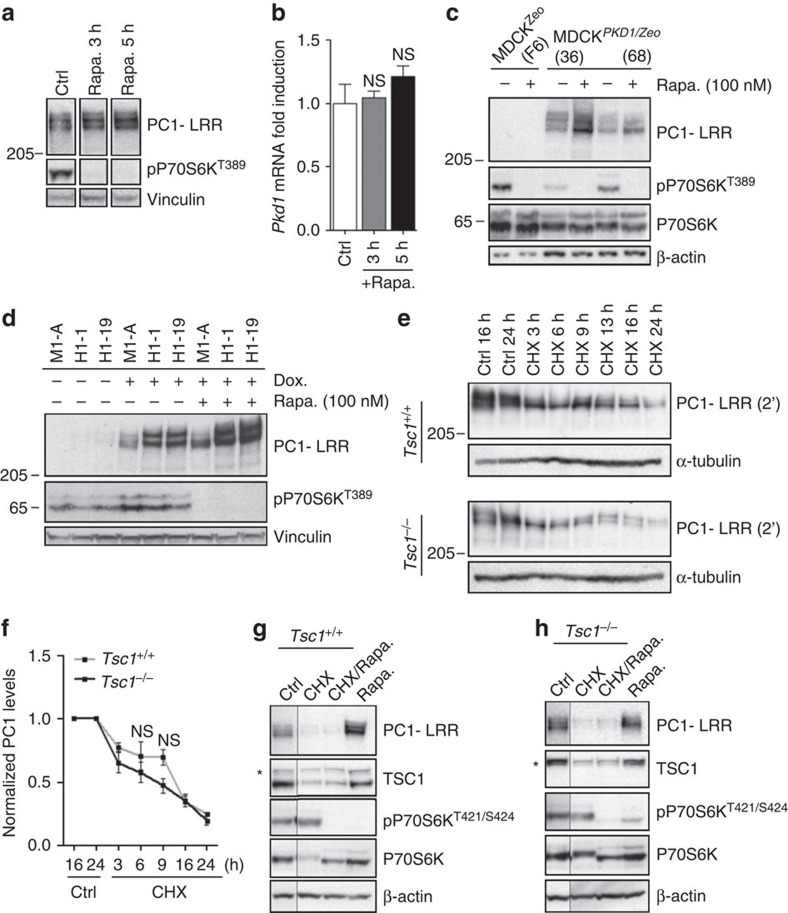
mTORC1 regulation of polycystin-1 requires protein neo-synthesis. (**a**) Immunoblot shows increased PC1-LRR levels in *Pkd1*^*Myc/Myc*^MEFs treated with 100 nM rapamycin for 3 and 5 h. Data are representative of two independent experiments. (**b**) qRT-PCR for *Pkd1* gene, nomalized towards *Arbp*, performed on mRNA extracted from cells in **a**. Data are shown as mean±s.d. ANOVA statistical analysis (NS, non significant) followed by Dunnett's multiple comparison test was performed. NS, non significant. (**c**) Western blot analysis in MDCK^Zeo^ (clone F2, F6) and *PKD1* overexpressing MDCK^*PKD1/*Zeo^ (clones G7/36, C8/68 and G3) cells shows PC-1 expression after 100 nM rapamycin for 24 h. Data are representative of two independent experiments. (**d**) Immunoblot showing PC1 levels in doxycicline-inducible MDCK cell line overexpressing the murine *Pkd1* (M1-A) and the human *PKD1* gene (H1-1, H1-19). Cells were treated 24 h with or without 100 nM rapamycin. (**e**) Time course analysis of PC-1 levels after protein neo-synthesis inhibition using 50 μM cycloheximide (CHX) in *Tsc1*^*+/+*^ and *Tsc1*^*−/−*^. (**f**) Graph shows quantification of three independent experiments. Data are shown as mean±s.e.m. ANOVA statistical analysis NS, non significant. (**g**,**h**) Treatment of *Tsc1*^*+/+*^ (**g**) and *Tsc1*^*−/−*^(**h**) MEFs in the presence of cycloheximide prevents the rapamycin ability to upregulate PC-1 levels. Data are representative of three independent experiments. Control lanes were run in the same blot and middle lanes were cut-out (Supplementary Fig. 7). *shows a non-specific band. ANOVA, analysis of variance.

From the above studies we conclude that mTORC1 regulates PC-1 expression levels and aimed at understanding how this regulation occurs. Quantitative real time analysis revealed no difference in the levels of *Pkd1* messanger RNA present in the *Tsc1*^*+/+*^ and *Tsc1*^*−/−*^ cells, nor in the *Tsc1* controls as compared to the *Tsc1*^*floxflox*^*:KspCre* kidneys, suggesting that downregulation does not occur at the transcriptional level (Supplementary Fig. 3a,b). We next asked whether rapamycin treatment might result in upregulation of the mRNA levels. Indeed, long-term treatment (24 h) did result in upregulation of the mRNA (Supplementary Fig. 3a,c). However, short-term treatment (3 and 5 h) was able to upregulate the protein levels (Fig. 3a), without affecting the mRNA levels (Fig. 3b). Furthermore, treatment with rapamycin enhanced the expression levels of PC-1 in two distinct sets of MDCK typeII cell lines stably transfected with the cDNA of human *PKD1* under the control of a viral promoter (Fig. 3c,d)^31^. These data taken together suggest that the regulation occurs primarily at the protein level, although long-term treatment with rapamycin might also affect the levels of transcript.

Next we asked if mTORC1 regulates PC-1 degradation levels. *Tsc1*^*+/+*^ and *Tsc1*^*−/−*^ MEFs were treated with Bortezomib (75 nM) to inhibit proteasomal degradation and we observed no effect on PC-1 expression levels in response to proteasomal inhibition (Supplementary Fig. 3d). Next we treated with Chloroquine (100 μM), which inhibits the lysosomes, the second major protein degradation pathway in cells (Supplementary Fig. 3e). This treatment did enhance PC-1 levels suggesting that this receptor is degraded via the lysosomes, but did not rescue the expression levels of PC-1 in *Tsc1*^*−/−*^ MEFs (Supplementary Fig. 3e). Similar results were generated using Bafilomycin to inhibit lysosomal degradation (Supplementary Fig. 3f). We next examined if mTORC1 regulates PC-1 neo-synthesis. We inhibited protein neo-synthesis with 50 μM cycloheximide in a time-course experiment both in *Tsc1*^*+/+*^ and *Tsc1*^*−/−*^ MEFs (Fig. 3e,f). Western blot analysis revealed that after 6 h of cycloheximide treatment PC-1 expression is approximately 50% of the total expression levels in both cell lines, even if the degradation curves appear slightly different within the first few hours, further supporting the notion that degradation rates are not different between control and *Tsc1*^*−/−*^ cells (Fig. 3e,f). After 24 h of cycloheximide treatment PC-1 appears almost totally degraded both in *Tsc1*^*+/+*^ and *Tsc1*^*−/−*^ fibroblasts (Fig. 3e,f). Notably, treatment of *Tsc1*^*+/+*^ and *Tsc1*^*−/−*^ MEFs with rapamycin (100 nM) in the presence or absence of 50 μM cycloheximide shows that the latter completely counteracts the rapamycin capability to enhance PC-1 expression levels (Fig. 3g,h). In conclusion, our data collectively demonstrate that mTORC1 activity negatively regulates the expression levels of PC-1 through a process requiring protein neo-synthesis and point to a possible role of mTORC1 in regulating *Pkd1* mRNA translation.

### Rapamycin induces expression of mature functional PC-1 in cilia

**Figure 4:**
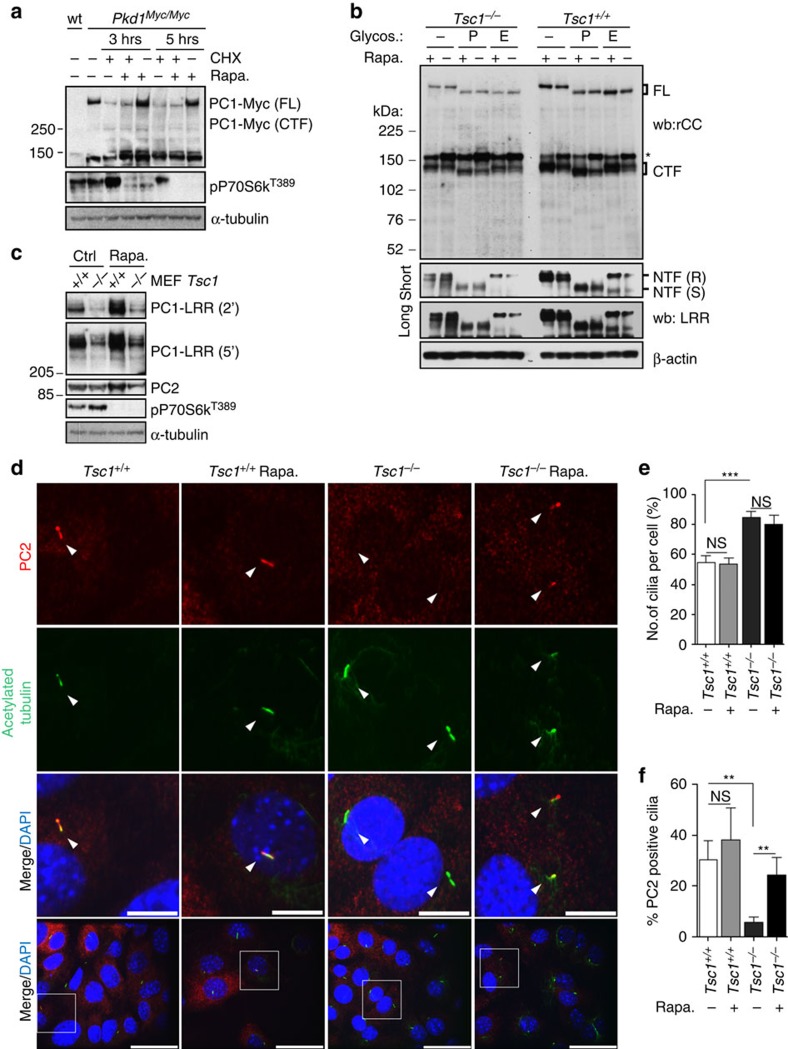
Protein neosynthesis is required for mTORC1 effect on PC-1 expression levels. (**a**) Western blot analysis with an anti-Myc antibody of *Pkd1*^*Myc/Myc*^ MEFs in the presence of cycloheximide shows that this prevents the capability of rapamycin (100 nM) to upregulate PC-1 levels. (**b**) Immunoblot detecting PC1 (rCC and LRR antibody) in MEFs treated with PNGaseF or EndoH. FL, full-length; NTF, N-terminal fragment; (R), EndoH-resistant; (S), EndoH-sensitive; CTF, C-terminal fragment. (**c**) Western blot detecting PC1 (LRR) and PC2 in *Tsc1*^*+/+*^ and *Tsc1*^*−/−*^MEFs treated in the presence or absence of 100 nM rapamycin. (**d**) Immunofluorescence staining of PC2 (red, arrows) and primary cilia (acetylated tubulin, green, arrows) performed in *Tsc1*^*+/+*^ and *Tsc1*^*−/−*^MEFs in the presence or absence of 100 nM rapamycin for 24 h. Scale bar, 10 μm (high magnification) and 40 μm (low magnification). Data are representative of three independent experiments. (**e**) Graph showing the quantification of the percentage of cilia per cell in *Tsc1*^*+/+*^ and *Tsc1*^*−/−*^MEFs, before and after 100 nM rapamycin for 24 h. Cells counted: *Tsc1*^*+/+*^ *n*=281, *Tsc1*^*−/−*^ *n*=437, *Tsc1*^*+/+*^ Rapa. *n*=166, *Tsc1*^*−/−*^ Rapa. *n*=304. Data are shown as mean±s.d. and are representative of three independent experiments. ANOVA statistical analysis (***P<0.0001) followed by Bonferroni's multiple comparison (compare selected pairs of columns) test was performed. NS, non significant. (**f**) Graph shows quantification of the percentage of PC2 positive cilia in in *Tsc1*^*+/+*^ and *Tsc1*^*−/−*^MEFs (number of cilia shown in **e**) before and after rapamycin treatment (100 nM for 24 h). Data are shown as mean±s.d. and are representative of three independent experiments. ANOVA statistical analysis (***P=0.0007) followed by Bonferroni's multiple comparison test (compare selected pairs of columns) was performed. NS, non significant; **P<0.01. ANOVA, analysis of variance.

PC-1 is a very-large polypeptide, which undergoes a number of post-translational modifications including glycosylation and cleavage^32^,^33^,^34^,^35^. We therefore asked whether the different isoforms of PC-1 might be differentially regulated by the mTORC1 pathway. As shown above, analysis of endogenous PC-1 using an antibody directed against the C-terminal tail of the protein revealed that both the uncleaved (FL-PC1) and the GPS-cleaved isoforms of PC-1 (CTF, C-terminal fragment and NTF, N-terminal fragment) are downregulated in *Tsc1*^*−/−*^ cells and both isoforms are sensitive to rapamycin treatment (Fig. 2e). Furthermore, treatment with rapamycin of MEFs carrying Myc-tagged endogenous PC-1 (ref. 24) further confirmed that both uncleaved and cleaved PC-1 are sensitive to rapamycin treatment (Fig. 4a). Interestingly, treatment in the presence of cycloheximide, even for short periods of time (3 and 5 h) prevented the rapamycin effect on PC-1 expression levels in these cells as well, in line with the idea that protein neo-synthesis is essential (Fig. 4a). Importantly, treatment with endoH revealed that the endoH-resistant, mature form of PC-1 is downregulated in *Tsc1*^*−/−*^ cells and is also sensitive to rapamycin treatment (Fig. 4b and Supplementary Fig. 4a,b). Since recent work^33^,^34^,^35^ has shown that this is the isoform of PC-1 which traffics to cilia and is likely essential for preventing renal cystogenesis, at least postnatally, our data implicate that mTORC1 can affect the expression levels of the functionally active form of PC-1 (ref. 33). This is also the isoform of PC-1 which allows trafficking of the PC-1/PC-2 complex to primary cilia^33^. Of interest, PC-2 expression levels are not sensitive to rapamycin treatment (Fig. 4c). Therefore, we used staining of PC-2 as a read-out to test the effect of mTORC1 on the capability of the PC-1/PC-2 complex to reach the primary cilium. We found that *Tsc1*^*−/−*^ cells have a reduced number of cilia which stain positive to PC-2 as compared to controls (Fig. 4d,f), even if they appear to have a slightly increased total number of cilia (Fig. 4e). Importantly, treatment in the presence of rapamycin, significantly increased the percentage of PC-2 positive cilia in *Tsc1*^*−/−*^ cells (Fig. 4f). Given that the total levels of the protein are unaffected (Fig. 4c), our data indicate that rapamycin quantitatively increases the amount of the PC-1/2 complex able to traffic to cilia.

### PC-1 downregulation mediates cystogenesis in *Tsc1* mutants

**Figure 5:**
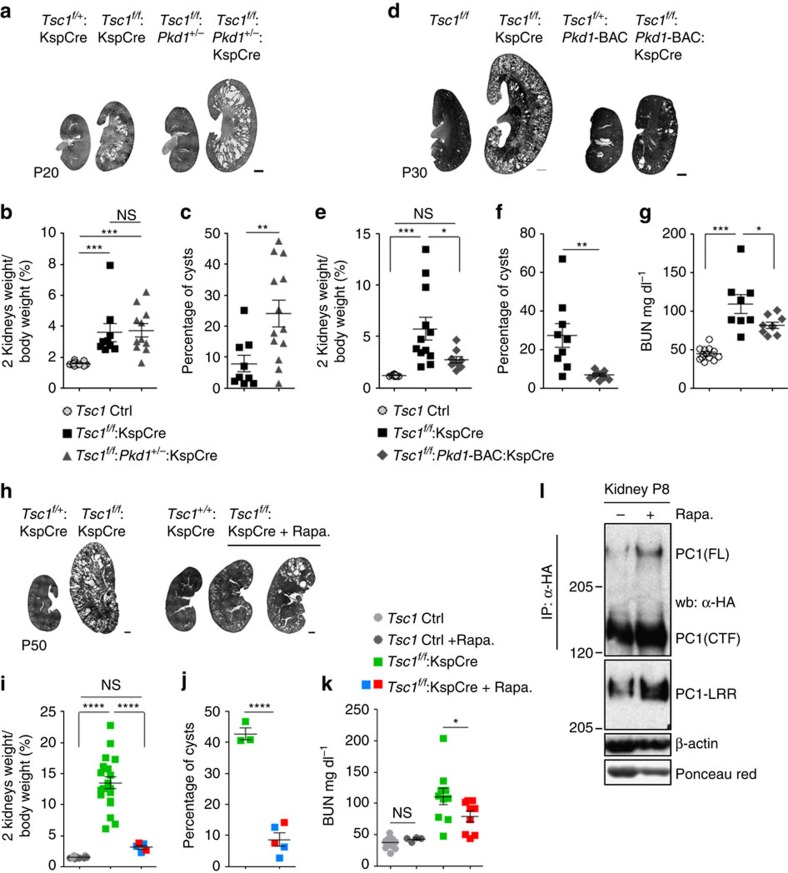
Downregulation of PC-1 in *Tsc1* mutant kidneys contributes to cyst formation. (**a**) H&E staining of kidney sections from *Tsc1*^*f/f*^*:KspCre*, *Tsc1*^*f/f*^*:Pkd1*^*+/*-^*:KspCre* and control mice at P20. Scale bar, 1,000 μm. (**b**) Graph shows the two kidneys over body weight ratio at P20 in *Tsc1*^*f/f*^*:KspCre* (*n*=9) and *Tsc1*^*f/f*^*:Pkd1*^*+/*-^*:KspCre* (*n*=13) compared with controls (*n*=18). Data are shown as mean±s.e.m. (**c**) Graph shows the cystic index in *Tsc1*^*f/f*^*:KspCre* (*n*=9) compared to *Tsc1*^*f/f*^*: KspCre:Pkd1*^*+/−*^ (*n*=13) mice at P20. Data are shown as mean±s.e.m. (**d**) H&E staining performed in P30 kidney sections from controls, *Tsc1*^*f/f*^*:KspCre* and *Tsc1*^*f/f*^*: KspCre:Pkd1*-BAC transgenic mice (*n*=5). Scale bar, 1,000 μm. (**e**) Graph shows the two kidneys over body weight ratio in controls (*n*=15), *Tsc1*^*f/f*^*:KspCre* (*n*=12) and *Tsc1*^*f/f*^*:Pkd1*-BAC*:KspCre* (*n*=9) transgenic mice. Data are shown as mean±s.e.m. (**f**) Cystic index analysis in kidneys at P30 of *Tsc1*^*f/f*^*: KspCre:Pkd1*-BAC transgenic mice (*n*=9) compared to *Tsc1*^*f/f*^*:KspCre* (*n*=9). Data are shown as mean±s.e.m. (**g**) Graph shows analysis of BUN at P30 in mice from figure (**e**) Data are shown as mean±s.e.m. (**h**) H&E staining on kidney sections from untreated or rapamycin (1 mg kg^−1^) treated *Tsc1*^*f/f*^:*KspCre* mice at P50. In green are shown the untreated *Tsc1*^*f/f*^*:KspCre* mice. Scale bar, 1,000 μm. (**i**) Graph shows the two kidneys weight over body weight ratio at P50 in controls (*n*=12) and *Tsc1*^*f/f*^*:KspCre* mice untreated (*n*=20) and after rapamycin treatment (*n*=5). (**j**) Graph shows the cystic index of one of two kidneys derived from mice treated in (**h**). Data are shown as mean±s.e.m. (**k**) Graph shows analysis of BUN at P50 in controls and *Tsc1*^*f/f*^*:KspCre* untreated (control *n*=19, cystic *n*=10) or treated with rapamycin (control *n*=4, cystic *n*=9). Data are shown as mean±s.e.m. (**l**) Immunoprecipitation of endogenous HA-PC-1 from P8 kidneys of *Pkd1*^*HA/HA*^mice, treated or not with 1 mg kg^−1^ rapamycin, followed by a western blot with anti-HA (FL, Full-length PC-1; CTF, C-terminal cleavage) or anti-LRR antibodies Data are representative of two independent experiments. ANOVA statistical analysis followed by Bonferroni's multiple comparison test was performed in **b**,**e**,**g**,**i**,**k**. Student's unpaired two-tailed *t*-test statistical analysis was performed in **c**,**f**,**j**. *P<0.05; **P<0.01;***P<0.001; ****P<0.0001; NS, non significant. ANOVA, analysis of variance.

Based on this we wondered if downregulation of PC-1 might be relevant in the context of the renal cystic phenotype observed in the *Tsc1*^*flox/flox*^*:KspCre* mice. We reasoned that if downregulation of PC-1 is involved in cyst formation, lowering the starting amount of PC-1 might enhance the phenotype in *Tsc1* mutants. To test this, we inter-crossed *Tsc1*^*flox/flox*^*:KspCre* with *Pkd1*^*+/−*^ mice, which do not manifest any cystic phenotype (Fig. 5a). Indeed, the resulting *Tsc1*^*flox/flox*^*:Pkd1*^*+/−*^*:KspCre* double mutants have a more aggressive renal cystic phenotype as compared with *Tsc1*^*flox/flox*^*:KspCre* mice (Fig. 5a). While the kidney/body weight did not change (Fig. 5b), the cystic index in the double mutants highlights a very significant increase in cystogenesis (Fig. 5c and Supplementary Fig. 5a–d), suggesting that the starting amount of PC-1 in these kidneys is a critical modifier of disease progression in the *Tsc1* mutants. Based on these data we hypothesized that downregulation of PC-1 in *Tsc1* mutants is an important driver of cyst formation. To formally test this hypothesis, we inter-crossed *Tsc1*^*flox/flox*^*:KspCre* mice with a murine line overexpressing three copies of a *Pkd1*-BAC clone (*Pkd1*^*F/H*^ transgenic line, Tg248)^36^. Overexpression of *Pkd1* indeed resulted in a much milder and delayed cystic phenotype (Fig. 5d) reduced kidney/body weight (Fig. 5e), a correction of the cystic index (Fig. 5f and Supplementary Fig. 6a) and an improved renal function (Fig. 5g). Of great interest, the *Pkd1* gene was not able to correct the columnar phenotype of the epithelium lining the cysts in *Tsc1* mutants, suggesting that additional factors must be altered in these epithelia and account for the different morphology (Supplementary Fig. 1g), in line with the fact that these mice eventually manifest cancerous lesions unlike the *Pkd1* mutants (unpublished data from L.D., M.P. and A.B.). A previous study has shown that inactivation of the *Pkd1* gene before P13 in the mouse results in an aggressive model of cystogenesis, whereas inactivation after P13 results in a much slower renal cystic phenotype^37^. Since our data demonstrate that cystogenesis in the *Tsc1*^*flox/flox*^*:KspCre* mice is influenced by the expression levels of PC-1, we reasoned that treatment of *Tsc1* mutant mice with rapamycin for a short period of time just to cover the critical window of time which determines the timing of a cystic manifestation in response to *Pkd1* inactivation should result in long-lasting beneficial effects on renal cystogenesis. Indeed, we treated *Tsc1*^*flox/flox*^*:KspCre* for a short time (P8–P20) followed by a release of the treatment for a longer time (P20–P50) and found that treatment for only 12 days to overcome the critical time point of P13 results in a very profound reversion of the renal cystic phenotype in these animals at P50 (Fig. 5h–j and Supplementary Fig. 6b) and improved renal function (Fig. 5k). Importantly, we used a mouse model carrying endogenous tagged PC-1 (*Pkd1*^*HA*^) (ref. 24) and found that treatment with rapamycin leads to enhanced expression of PC-1 in the kidney, further confirming that mTORC1 regulates PC-1 expression levels *in vivo* (Fig. 5l).

## Discussion

**Figure 6:**
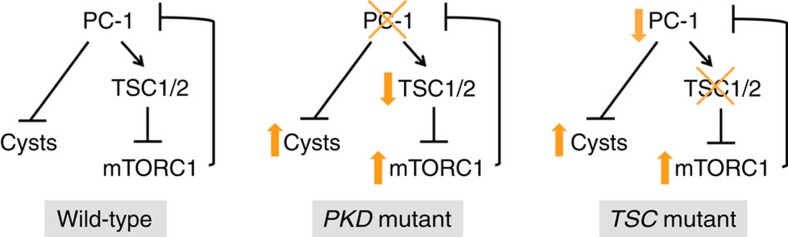
Proposed model of cross regulation of the PKD and TSC genes. Schematic summary of the proposed model. PC-1 inhibits the mTORC1 pathway by acting on the TSC genes (previous work^3^,^4^), whereas mTORC1 acts in a negative feedback loop to downregulate PC-1 expression levels.

We conclude that downregulation of PC-1 plays a key role in the cystogenesis observed in *Tsc1* mutants. In addition, given the different phenotype observed in the *Pkd1* and *Tsc1* mutant kidneys our data show that likely PC-1 protection from cystogenesis is mediated through mTORC1-independent mechanisms (Fig. 6). However, mTORC1 upregulation in response to *Pkd1* inactivation likely contributes to increased proliferation, justifying the beneficial effects observed upon treatment with rapamycin in several PKD models^8^.

PC-1 expression levels are finely regulated and its downregulation below a critical threshold of expression or activity causes cyst formation in humans and mice^35^,^38^,^39^,^40^. In the model proposed in this study PC-1 inhibits mTORC1, which in turn downregulates PC-1 levels, in a feedback loop, which is ultimately acting as a positive feedback on mTORC1 activity (Fig. 6). Our data contribute to explaining the renal cystic phenotype observed upon inactivation of *Tsc* genes in the kidney, but more importantly they potentially explain the severe cystic phenotype observed in individuals affected by the *TSC/PKD* contiguous genes syndrome^19^. In this case, each gene is halved and any perturbation of the system causing a minimal drop of activity in one or the other of the two gene products would result in a self-perpetuating circle causing progressive upregulation of mTORC1 and downregulation of PC-1 (Fig. 6). Importantly, our data suggest that rapamycin or other mTOR inhibitors might be more effective in these patients, or in any renal cystic disease in which some level of activity of the *PKD1* gene is maintained^39^,^41^. The severe cases of ADPKD (carrying truncating mutations) might not be sensitive to rescue through the mechanism proposed here^41^.

One implication of our results is that it might be interesting to revisit some of the studies generated by the use of everolimus or sirolimus in clinical trials^16^,^17^, by stratifying the treated patients on the basis of their inherited genetic germline mutation. In fact, a landmark study has shown that there is a genotype–phenotype correlation when analysing the renal outcome in patients carrying *PKD1* mutations: those individuals carrying germline missense mutations have a better outcome than those individuals carrying truncating mutations^41^. These results suggest that the *PKD1* missense variants retain some of their function. Further to this, previous studies have shown that occasionally individuals can carry hypomorphic mutations in the *PKD1* gene, which do not cause any or a very mild renal manifestation when carried in heterozygosity, but show a marked renal cystic phenotype when carried in homozygosity^39^. These data strongly suggest that the type of mutation carried by the *PKD1* gene can cause a graded effect on the polycystin-1 protein function that can range from very mild to very severe. Based on our proposed model we would predict that individuals carrying a missense or a hypomorphic mutation should be more sensitive to the treatment with any analogue of rapamycin because upregulation of a PC-1 protein with reduced function might be sufficient to bring the level of activity above threshold, thus correcting cystogenesis. Importantly, our study suggests that discontinuation of the drug might apply in all these cases, possibly reducing any side effect.

Further to this, previous studies have suggested that PC-1 downregulation is central to the renal cystic phenotype in ARPKD^36^,^42^. Chaperones able to facilitate PC-1 trafficking might be one possible approach to restore its function^43^. Based on our data, we would predict that rapamycin or any inhibitor of mTORC1 may act through the mechanism proposed in this study and be effective in ARPKD as well. Obviously it would not be conceivable to administer any of the current compounds inhibiting mTOR in infants or during pregnancy and the short-term implications of our studies for this specific disease are certainly less immediate. However, if humans are sensitive to a critical developmental window of PC-1 expression similar to what has been observed in mice^37^, one could think to achieve a long-term beneficial effect in this devastating human condition by correcting PC-1 expression levels during the fetal or neonatal life. Our study shows that the regulation of PC-1 by mTORC1 occurs at the post-transcriptional level and likely at the protein synthesis level. It will be essential to further characterize the molecular mechanism underlying this regulation and to identify the key molecules involved. This might further open therapeutical opportunities allowing to achieve PC-1 upregulation without administration of rapalogues.

In sum, our data uncover a novel, unanticipated reciprocal regulation of PC-1 and mTORC1, which revisits the role of mTORC1 in renal cystogenesis, opening new perspectives for therapy.

## Methods

### Antibodies and inhibitors

For western blot analysis we used antibodies against pP70S6K Thr389 (#9205 1:1,000), pP70S6K Thr421/Ser424 (#9204 1:1,000), pS6Rp Ser235/236 (#2211 1:5,000), pS6Rp Ser240/244 (#2215 1:10,000), S6RP (# 2217 1:1,000), TSC1 (#4906 1:1,000), β-Catenin (#9562 1:1,000) and Myc-Tag (#2272) obtained from Cell Signaling Technology. HA antibody (#11867423001, 1:500) was from Roche. P70S6K C-18 (#sc-230 1:1,000), PC1-LRR 7E12 (#sc-130554 1:1,000), PC2 D3 (#sc-28331 1:500), Ubiquitin P4D1 (#sc-8017 1:10,000) and DAPI (# sc- 3598 1:5,000) were from Santa Cruz Biotechnology. The rat monoclonal antibody (rCC) against the C-terminal tail of mouse PC1 was described in Kurbegovic *et al*.^30^ Antibodies to detect LC3 NB100 (#2331 1:500) from Novus Biologicals, β-actin (#A5441 1:25,000) and α-tubulin (#T5168 1:25,000) from Sigma Aldrich, Vinculin V284 (#05-386 1:15,000) was obtained from Millipore. Horseradish peroxidase (HRP)-conjugated secondary antibodies were from GE Healthcare, anti-rabbit HRP linked (# NA934V) and anti-mouse HRP linked (# NXA931). For immunofluorescence staining LTL (Fluorescin Lotus Lectin) #FL-1321 and DBA (Rhodamine Dolichos Biflorus Agglutinin) #RL-1032 were obtained from Vector Laboratories and used 1:100, Acetyl-α-Tubulin (Lys40) (D20G3) (#5335) was from Cell Signaling Technology and used 1:1,000. For immunohistochemistry we used the Ki67 antibody (NCL-Ki67p) concentrated 1:200 and obtained from Novocastra.

Rapamycin was obtained from LC Laboratories (#R-5000) dissolved in DMSO and used at a final concentration of 100 nM (or other) for cell treatment. Bortezomib (#B1408) from LC Laboratories was dissolved in DMSO and used at a final concentration of 25, 50 and 75 Nm for 16 h. Chloroquine (#C-6628, Sigma) was dissolved in water and used for 18 h at 25, 50 and 100 μM final concentration. Cycloheximide (#C-7698, Sigma) dissolved in ethanol and used at 50 μM concentration. Bafilomycin A1 (#B1793) was obtained from Sigma, dissolved in DMSO and used at a final concentration of 50 Nm. Torin 1 (#4247) was obtained from Tocris, dissolved in DMSO and used 300 nM.

### Cell Cultures and treatments

For rapamycin treatment MEFs^9^, MDCK and mIMCD epithelial cells^31^ were cultured sub-confluent in DMEM (Gibco) supplied with 10% serum (Euroclone) and 1% Pen/Strep (Gibco). Before treatment cells were serum starved overnight using DMEM supplied with 0.4% serum, 1% Pen/Strep. For bortezomib, chloroquine and cycloheximide treatment MEFs were plated at high confluence.

For the glycosylation assay we used New England Biolabs' enzymes according to their instruction. Cell lysates were denatured using glycoprotein denature buffer for 1 min at 95 °C and then quickly chilled on ice. The denatured glycoprotein was incubated with PNGaseF or EndoH for 1 h at 37 °C (ref. 33).

### Generation of *Tsc1*-silenced mIMCD cells

Pre-screening of shRNAs targeting murine *Tsc1* was performed as follows: 500,000 mIMCD cells were seeded and 24 h later transduced with viral vectors expressing shRNA encoding scrambled (shScr) sequences (MISSION TRC2 Control Transduction Particle puro Non-Target shRNA 4,1 × 10^7^ TU ml^−1^ SHC202V from SIGMA) or six different *Tsc1*-targeting shRNAs sequences (shTSC1 A-F, MiSSION Lentiviral Transduction Particles SHCLNV from SIGMA, batch A TRCN0000113949 1,8 × 10^7^ TU ml^−1^, batch B TRCN0000238186 2,0 × 10^7^ TU ml^−1^, batch C TRCN0000238187 2,0 × 10^7^ TU ml^−1^, batch D TRCN0000238188 1,5 × 10^7^ TU ml^−1^, batch E TRCN0000238189 1,5 × 10^7^ TU ml^−1^ and batch F TRCN0000244252 1,6 × 10^7^ TU ml^−1^) using multiplicity of infection 1 and 2. 48 h after transduction cells were collected, lysed and analysed by SDS–polyacrylamide gel followed by western blot analysis using anti-TSC1, anti-pP70^T389^, anti-pS6Rp^S235-236^ and anti-α tubulin antibodies.

For stable transduction, 500,000 mIMCD cells were seeded into six-well plates and grown in DMEM (Invitrogen), supplemented with 10% v/v fetal bovine serum and 1:100 penicillin 5,000 U ml^−1^ per 5000 μg ml^−1^ streptomycin solution (Invitrogen). mIMCD cells were transduced with viral vectors expressing shRNA encoding scrambled sequences (shScr) or the selected Tsc1-targeting shRNA sequence (shTSC1) under puromycin selection, using MOI 2. 48 h after transduction, cells were splitted and medium containing 1 μg ml^−1^ puromycin (Invitrogen) was added. Untransduced cells were treated with the same puromycin concentration to establish the maximal toxicity of puromycin. After 6 days no untransduced cells survived, and selection of puromycin-resistant clones was concluded. Clones were collected as pools containing several different selected clones. Resistant cells were analysed for *Tsc1* expression levels as above. Next, resistant clones were sub-cloned by limiting dilutions and 20 sub-clones selected for shTSC1C, 14 clones for shTSC1F and 10 clones for shScr. At confluency cells were analysed for *Tsc1* and mTORC1 pathway-related protein (pP70 and pS6Rp) expression levels and phosphorylation. Two clones carrying high silencing levels (F5 and F8) and two control clones (Sc4 and Sc8) were selected for further use.

### Generation of *Pkd1* and *Tsc1* mice

To generate *Pkd1*^*f/−*^*:KspCre* mice we crossed *Pkd1*^*f/f*^(ref. 24) and *Pkd1*^*+/−*^*:KspCre* mice. To generate *Tsc1*^*f/f*^*:KspCre* and *Pkd1*^*f/f*^*:KspCre* mice we inter-crossed *Tsc1*^*f/+*^*:KspCre* and *Pkd1*^*f/+*^*:KspCre* mice, respectively. To generate *Tsc11*^*f/−*^*:KspCre* mice we crossed *Tsc1*^*f/f*^ and *Tsc1*^*f/-:*^*KspCre* mice that were in a mixed genetic background (fourth generation of backcrosses in C57BL6 pure genetic background). To generate *Tsc1*^*f/f*^*:Pkd1*-BAC*:KspCre* mice we crossed *Tsc1*^*f/+*^*:KspCre* with *Tsc1*^*f/+*^*: Pkd1*-BAC*: KspCre*. *Pkd1*-BAC (Tg.248) were described elsewhere^36^. All the mice used in these experiments were in pure C57BL6 genetic background (backcrossed over nine times). Animals used were half males and half females. For rapamycin (LC Laboratories) treatments, we intraperitoneally injected rapamycin in DMSO in NaCl or vehicle alone daily to the mothers from P2/3 until P10 at 1 mg per kg body weight. Starting from P10 we start treating directly the pups by intraperitoneal injections of rapamycin at 1 mg per kg body weight or vehicle alone. All animal care and experimental protocols were conducted after approval of a specific protocol (IACUC-548) by the institutional care and use ethical committee at the San Raffaele Scientific Institute.

### Histology, immunofluorescence and immunohistochemistry

For histological analysis mice were sacrificed at the given time point, kidneys were collected, weighted and fixed in 4% Paraformaldehyde over night at 4 °C. For rapamycin treated mice, before collecting kidneys, mice were anaesthetized and perfused in PBS. Fixed kidneys were processed through a sucrose (Sigma) gradient in PBS from 10 to 30%, treated with 10% glycerol, 30%sucrose, PBS and finally embedded in Killik (Bio Optica) for frozen sections. Kidney sections were air dried, rehydrated in PBS, stained for 2 min in 1:10 Harris Hematoxylin (Sigma), washed in abundant water, stained 30 s in eosin G (Bio Optica) and washed again. After staining, kidney sections were dehydrated through an alcohol gradient and then mounted in DPX (Sigma).

For immunofluorescence staining of DBA and LTL positive tubules 14 μm kidney sections were washed in PBS, fixed 10 min in 4% paraformaldehyde and permeabilized in 0,1% Triton x-100,PBS. We blocked sections in 5% Normal Goat serum (Sigma), 3% BSA, PBS for 1 h room temperature, incubated overnight at 4 °C with primary antibodies diluted in blocking solution as described, washed with PBS, the nucleus was stained with DAPI and mounted with mowiol (Sigma). Images were obtained using UltraView spinning disk confocal microscope (PerkinElmer) with a Plan Apochromat 63X/1.4 oil immersion or a × 20 objective using the UltraVier ERS acquisition software.

For immunohistochemistry of Ki67 positive cells, frozen kidney sections were air dried and washed in PBS. Endogenous peroxide was blocked for 10 min with 0.5% v.v. hydrogen peroxide in MetOH. For antigen unmasking sections were boiled in 10 mM citrate buffer Ph 6.0. Primary antibody was incubated O/N in 3% BSA, 5% NGS in PBS. The secondary antibody and the DAB^+^ developing solution were from Dako (K3468). H&E staining was used to vizualize nuclei, sections were dehydrated and mounted in DPX.

### Cystic index analysis

To quantify the number of cysts, we obtained longitudinal sections from the inner part of the kidney, performed H&E staining and then get × 4 images (Nikon Eclipse E600 microscope, Nikon Digital Camera DXM1200, ACT-1 software) for each kidney section. We used Image J programme (http://rsb.info.nih.gov/ij/) to quantify the surface, expressed in pixels, covered by cysts in each kidney. We used a cutoff of 2,000 pixels as the minimum surface that a cyst should have to be included in the analysis. We next calculated the ratio between the obtained cystic area and the area of the entire kidney section and expressed it as the percentage of cysts for the given kidney.

### Real-time PCR analysis

We isolated total RNA from plated cells using the RNAspin Mini kit (GE Healthcare). Total RNA from kidneys was isolated using the RNA-Isol Lysis reagent (5 Prime) according to the manufacturer's instructions. We obtained cDNA from using Oligo(dt)_12-18_ primers (#18418012) and Superscript II Reverse Transcriptase (#18064014) from Life Technologies. For reverse transcription of RNA extracted from kidneys, we used Oligo(dt)_15_ primers (#C110B) and ImProm-II Reverse Transcriptase (#A3802) from Promega. We performed quantitave real-time PCR analysis on duplicate using SYBR Green I master mix (#04707516001,Roche) on LightCycler 480 Instrument (Roche). We used the following primers for qRT-PCR: *Pkd1* FW 5′- GGCGGCTTTGTGATTTGTAT -3′, *Pkd1* RV 3′- ACCTGAATCGGGGGATAAAC -5′, Beta actin FW 5′- AGAAAATCTGGCACCACACC -3′, Beta actin RV 3′- CAGAGGCGTACAGGGATAGC -5′. *Arbp* FW 5′- CTTCATTGTGGGAGCAGACA -3′ and *Arbp* RV 3′- TTCTCCAGAGCTGGGTTGTT -5′.

### Western blot analysis

For western blot analysis cells or kidneys were lysed in lysis buffer solution of 150 mM NaCl, 20 mM Na_4_HPO_4_/NaH_2_PO_4_, 10% glycerol, 1% Triton X-100 (pH 7.2), complete protease inhibitors (Roche) and phosphatase inhibitors (1 mM final concentration of glycerophosphate, sodium orthovanadate and sodium fluoride). Total lysates were then quantified with Biorad Protein Assay (Biorad) and Laemmli buffer at a final concentration × 2 was added to the samples. Proteins were next resolved in 3–8% Tris-Acetate gels (Life Technologies) and then transferred onto polyvinylidene fluoride membranes (Millipore). We then blocked membranes with 5% milk in Tris-buffered saline, Tween 20 (TBS-T). All the primary antibodies for western blot analysis were diluted in 3% BSA (#A7906, Sigma), TBS-T. HRP-conjugated secondary antibodies were diluted 1:10,000 (or more if necessary) in 5% milk, TBS-T and detection was made with ECL (#RPN2106, GE) alone or supplied with 10% SuperSignal West Femto (#34095,Thermo Scientific) when necessary.

### Immunoprecipitation studies

To immunopecipitate PC-1 HA, P8 kidneys were collected from a mouse model carrying endogenous HA tagged PC-1 (*Pkd1*^*HA*^)^24^, treated or not treated with 1 mg kg^−1^ rapamycin from P4 to P8. Kidneys were lysed in lysis buffer solution with 1% Triton X-100 and equal amounts of total lysates were incubated over night at 4 °C in rocking conditions with anti-HA-high affinity matrix (Roche). After several washes with lysis buffer, beads were resuspended in loading laemmli buffer and proteins resolved in 3–8% Tris-acetate gradient gels.

## Additional information

**How to cite this article:** Pema, M. *et al*. mTORC1-mediated inhibition of polycystin-1 expression drives renal cyst formation in tuberous sclerosis complex. *Nat. Commun.* 7:10786 doi: 10.1038/ncomms10786 (2016).

## Supplementary Material

Supplementary Information Supplementary Figures 1-7.
