# Supplementary material for: mTORC1-mediated inhibition of polycystin-1 expression drives renal cyst formation in tuberous sclerosis complex
Source: Nat Commun. 2016 Mar 2;7:10786. doi: 10.1038/ncomms10786 (PMC4778067; doi:10.1038/ncomms10786)
Supplement: Supplementary Information — Supplementary Figures 1-7. [file ncomms10786-s1.pdf]

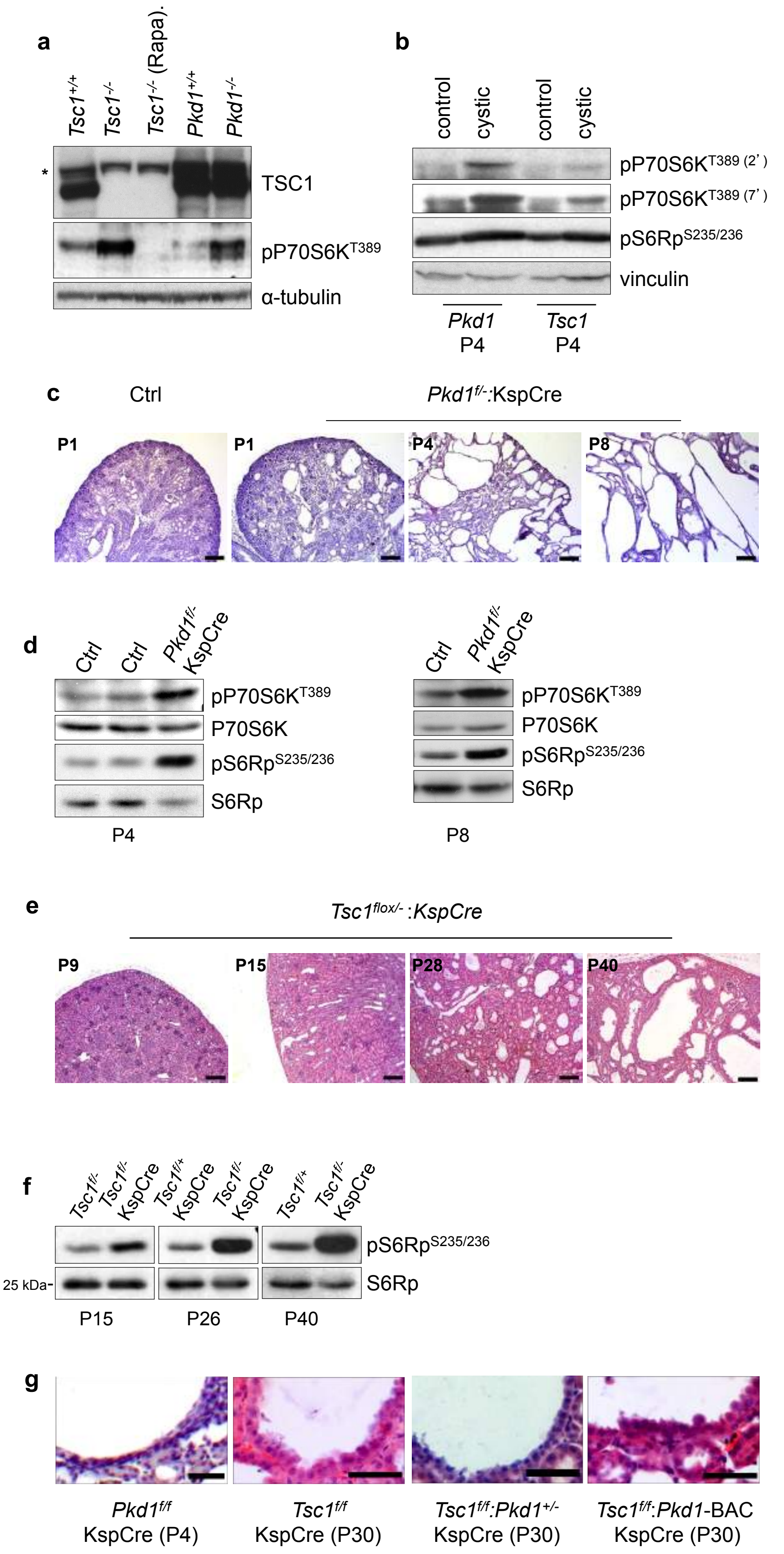

Supplementary Figure 1. Comparative analysis of *Pkd1*$^{f/-}$*:KspCre* and *Tsc1*$^{f/-}$*:KspCre* (**a**)Western blot analysis performed in *Tsc1*$^{+/+}$ and *Tsc1*$^{-/-}$ cells versus *Pkd1*$^{+/+}$ and *Pkd1*$^{-/-}$ MEFs. The mTORC1 activation status is evidenced. (*) indicates an aspecific band. (**b**) Western blot analysis shows the activity of the mTORC1 cascade in kidneys from *Pkd1*$^{f/f}$*:KspCre* and *Tsc1*$^{f/f}$*:KspCre* mice, indicated as cystic, versus controls at P4. (**c**) H&E staining of kidney sections from P1, P4 and P8 *Pkd1*$^{f/-}$*:KspCre* and control. Images are representative of at least n= 10 mice analyzed in the laboratory. Scale bar, 200µm. (**d**) Western blot analysis performed in total kidney lysates shows mTORC1 activation status in *Pkd1*$^{f/-}$*:KspCre* versus controls at P4 and P8. Data are reperesentative of at least three indipendent experiments (**e**) H&E staining of kidney sections from *Tsc1*$^{f/-}$*:KspCre* kidneys at P9, P15, P28 and P40. Scale bar, 200µm. (**f**) Immunoblots in total kidney lysates from *Tsc1*$^{f/-}$*:KspCre* and controls shows mTORC1 pathway activity at P15, P26 and P40. Data represent one experiment for each time point. (**g**) Histological analysis reveals a different morphology of the epithelia lining the cysts in the indicated genotypes at the indicated time points. Scale bar: 50µm

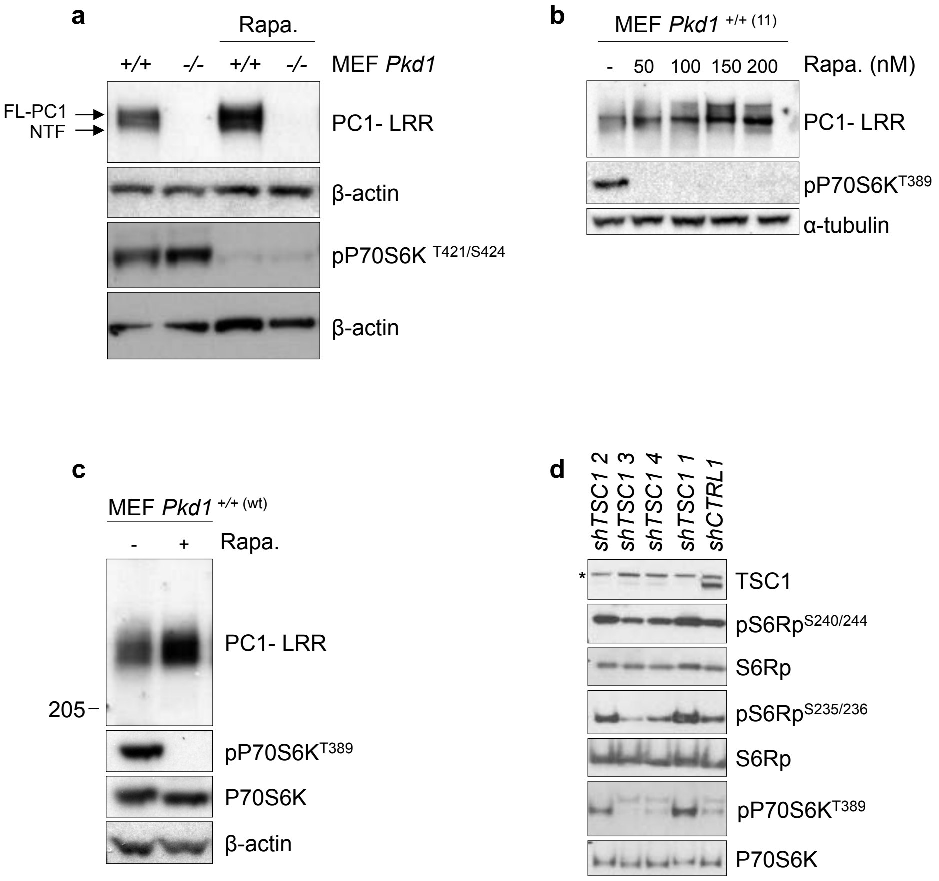

Supplementary Figure 2. mTORC1 regulates PC-1 expression levels. (**a**) Western blot analysis in *Pkd1*$^{+/+}$ and *Pkd1*$^{-/-}$ fibroblasts detects endogenous PC-1 before and after treatment with Rapamycin 100nM for 24 hours (n=5). (**b**) Western blot analysis of PC-1 in *Pkd1*$^{+/+}$ MEFs cultured for 24 hours in the presence of 50,100,150 and 200 nM rapamycin. (n=1). (**c**) 24 hours treatment with 100nM rapamycin of an additional *Pkd1*$^{+/+}$ MEFs line shows increased PC-1 levels. (**d**) Several clones selected after infection with the shRNA against the *Tsc1* gene were isolated and analyzed for *Tsc1* downregulation and mTORC1 levels. The clones (sh2 and 1) that showed higher level of inactivation showed upregulation of mTORC1 and were used for further studies. (*) indicates an aspecific band.

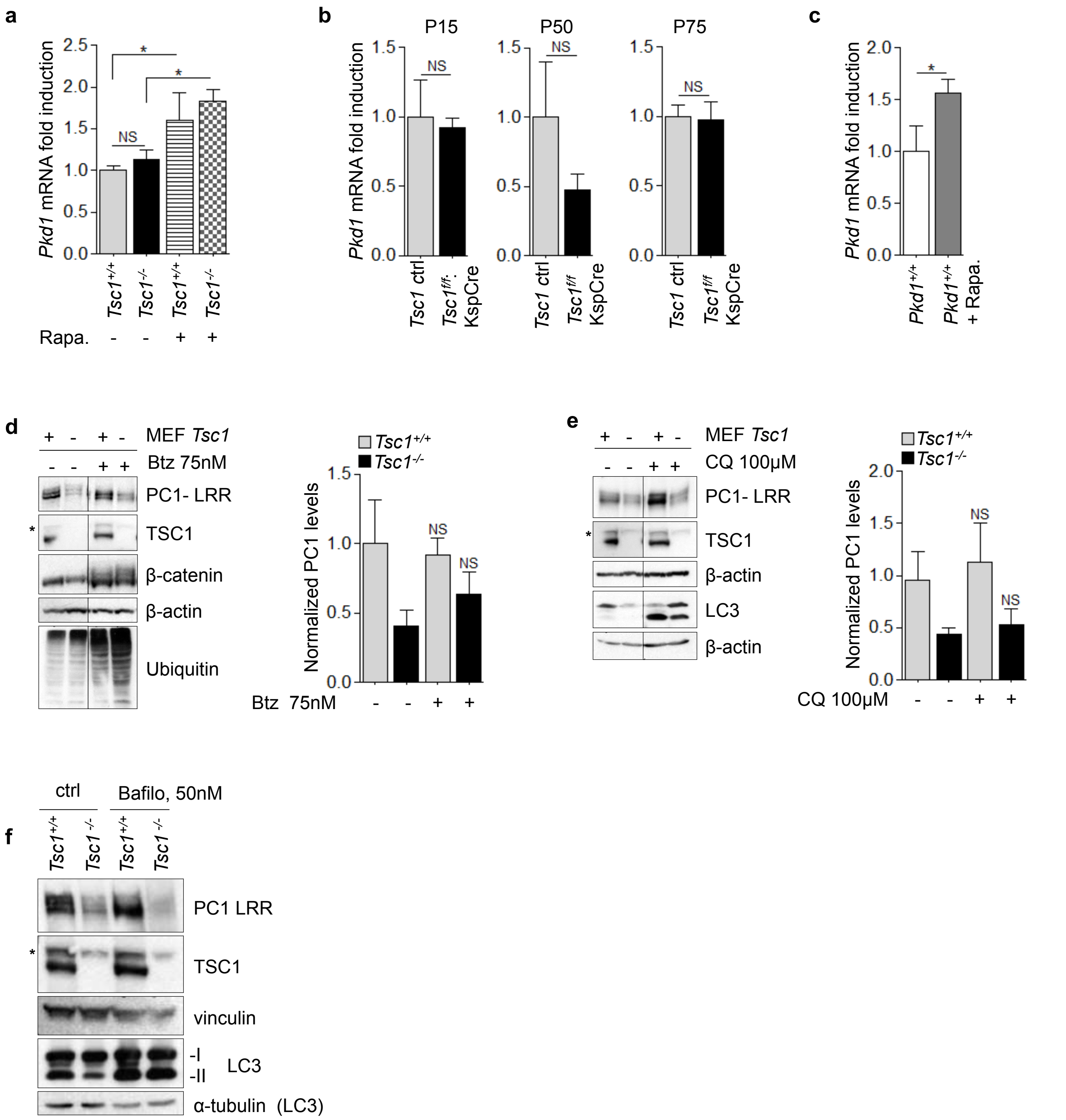

Supplementary Figure 3. Downregulation of PC-1 by mTORC1 is not at the transcriptional or degradative level. (**a**) qRT-PCR analysis shows *Pkd1* mRNA expression levels, normalized toward *beta actin* gene, in *Tsc1*$^{+/+}$ and *Tsc1*$^{-/-}$ MEFs before and after 100nM rapamycin treatment for 24 hours. Data are shown as mean±SEM. ANOVA statistical analysis followed by Bonferroni's multiple comparison test (compare selected pairs of columns) was performed. NS, non significant; *p<0.05. (**b**) Graph shows *Pkd1* mRNA expression levels, normalized on *Arbp*, analyzed by qRT-PCR. Total mRNA was extracted from P15, P50 and P75 (n=6 each performed in experimental duplicate) *Tsc1*$^{f/f}$*:KspCre* kidneys versus controls (n=6). Statistical analysis was performed using the Student's two-tailed unpaired *t*-test. NS, non significant; p=0.78 for P15 mice, p=0.23 for P50 mice and p=0.30 for P75 mice. F-test was used to compare variances.p=0.0120 (*) for 15, p=0.0129(*) for P50 ; p=0.19 for P75 (**c**) qRT-PCR analysis shows *Pkd1* mRNA expression levels, normalized toward *beta actin* gene, in *Pkd1*$^{+/+}$ cells in the presence or absence of 100nM rapamycin for 24 hours; n=4 for each condition. Data are shown as mean±SEM. Student's one tail, paired t-test was performed. *p=0.0155. (**d**) Western blot in *Tsc1*$^{+/+}$ and *Tsc1*$^{-/-}$ fibroblasts treated with bortezomib (Btz, 75nM). (*) indicates an aspecific band. Graph shows quantifications of immunoblots from (n=3) independent experiments. Data are shown as mean ±SEM. ANOVA statistical analysis (***p=0.0004) followed by Bonferroni's multiple comparison test was performed. NS, non significant. (**e**) Western blot in *Tsc1*$^{+/+}$ and *Tsc1*$^{-/-}$ fibroblasts treated with chloroquine (CQ, 100μM) for 16 hours. (*) indicates an aspecific band. Graph shows quantification of immunoblots from (n=4) independent experiments. Data are shown as mean±SEM. ANOVA statistical analysis (ns, p=0.0861) followed by Bonferroni's multiple comparison test was performed. NS, non significant (**f**) Western blot shows *Tsc1*$^{+/+}$ and *Tsc1*$^{-/-}$ MEFs before and after 24 hours treatment with 50nM Bafilomycin. PC1-LRR protein levels are shown. (*) indicates an aspecific band.

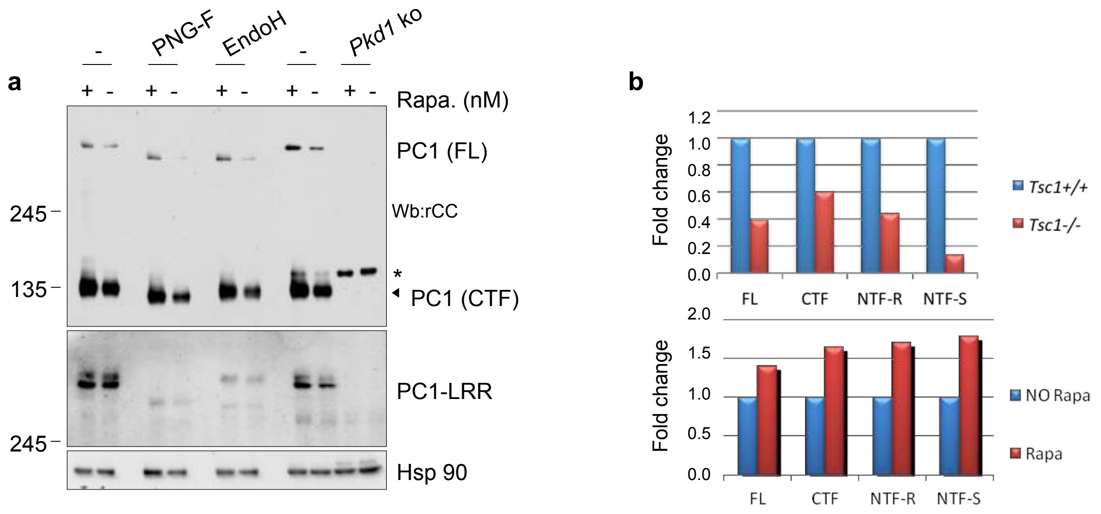

Supplementary Figure 4. All isoforms of PC-1 are equally affected by mTORC1 regulation. (**a**) Immunoblot detecting PC1 (rCC and LRR antibody) in MEFs treated with PNGaseF or EndoH. (*) indicates an aspecific band of PC1 (CTF), while arrowhead indicates the specific band. (**b**) Quantification of the individual bands shown in the blot in Figure a are represented expressed in the fold changes over the controls. All bands change in a very similar manner in the different experiments. Importantly, the NTF-R (endoH resistant, going to the plasma membrane) is downregulated in the *Tsc1*$^{-/-}$ cells and is upregulated upon rapamycin treatment, similarly to all other bands.

a

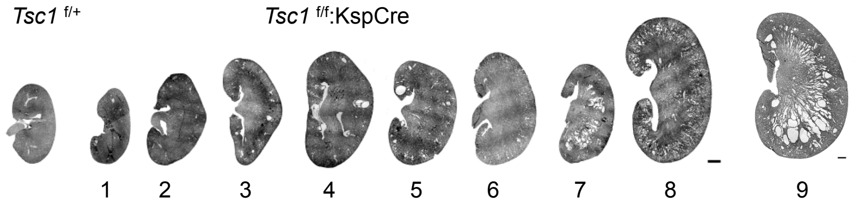

b

Tsc1$^{f/f}$:KspCre n°	1	2	3	4	5	6	7	8	9
% cysts	1.48	1.49	3.32	2.08	7.03	3.35	13.87	13.03	25.15
2 kidneys weight (gr)	0.15	0.23	0.30	0.26	0.32	0.23	0.22	0.70	0.40
body weight (gr)	5.00	9.90	11.10	9.80	9.40	9.50	7.70	8.90	9.10

c

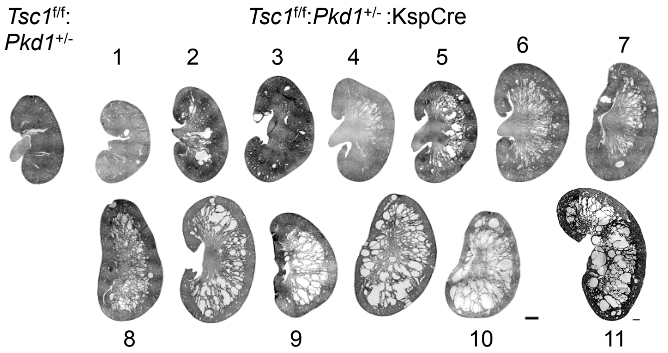

d

Tsc1$^{f/f}$:*Pkd1*$^{+/-}$: KspCRe n°	1	2	3	4	5	6	7	8	9	10	11
% cysts	1.45	14.28	6.00	9.16	18.86	17.76	15.27	25.28	35.26	47.37	44.04
2 kidneys weight (gr)	0.24	0.21	0.27	0.26	0.12	0.46	0.32	0.46	0.29	0.38	0.55
body weight (gr)	9.70	7.10	9.40	9.90	7.70	9.8	10.3	7.5	7.00	8.70	9.80

Supplementary Figure 5. Analysis of all individual kidneys of *Tsc1* mutants and *Tsc1/Pkd1* double mutants. (**a**) H&E staining of kidney sections from P20-21 control and *Tsc1*$^{f/f}$*:KspCre* (n=9). Scale bar, 1000µm. (**b**) The table summarizes the values of the percentage of cysts, two kidneys and body weight for samples from 1 to 9 shown in figure (**a**). (**c**) H&E staining of kidney sections from P20-21 control and *Tsc1*$^{f/f}$*:Pkd1*$^{+/-}$*:KspCre* mice (n=13). Scale bar, 1000µm. (**d**) The Table summarizes the values of the percentage of cysts affecting *Tsc1*$^{f/f}$*:Pkd1*$^{+/-}$*:KspCre* kidneys, the two kidneys and body weight are shown for sample 1 to 11 presented in figure (**c**).

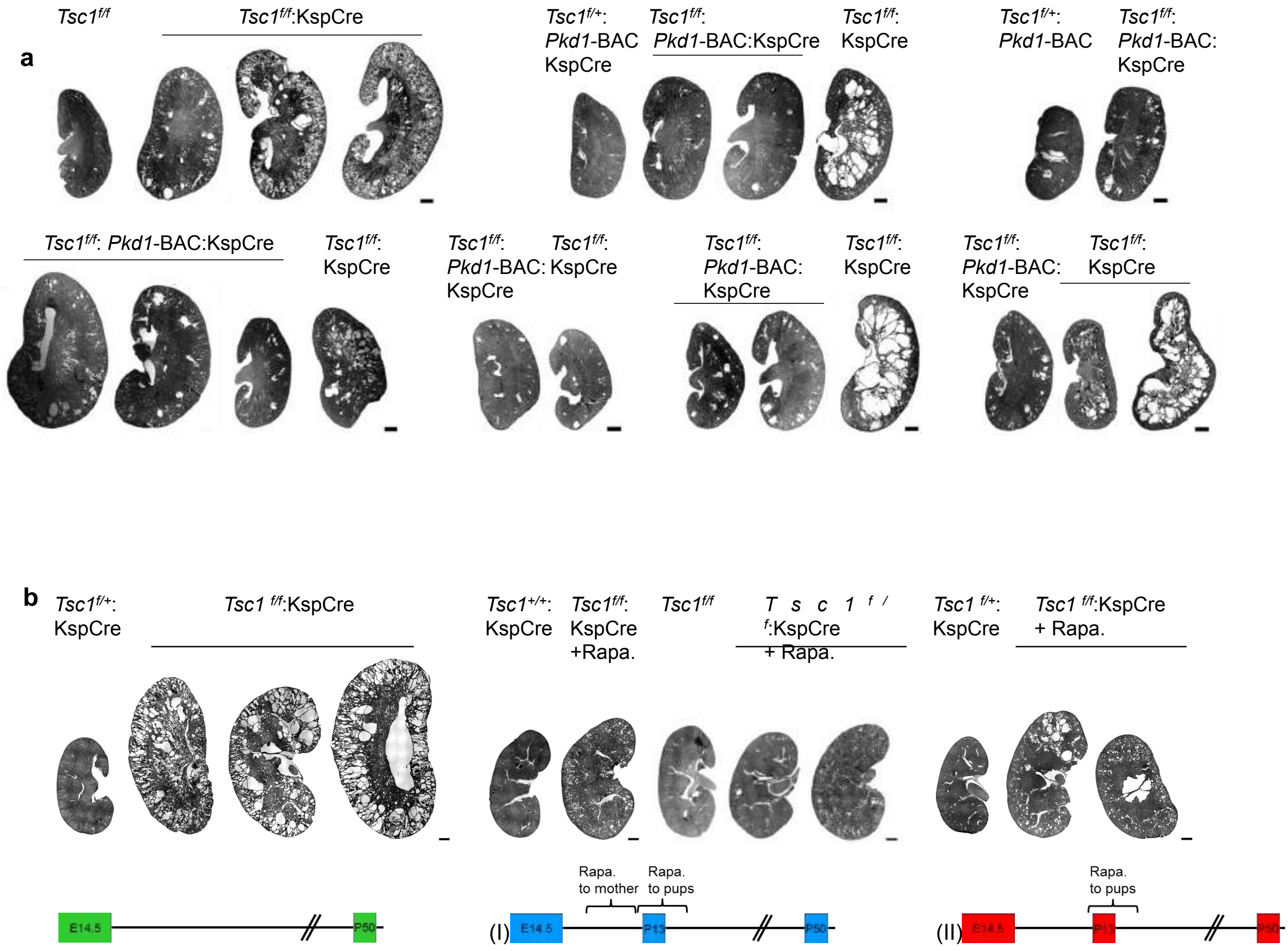

Supplementary Figure 6. Analysis of all individual kidneys of *Tsc1* mutants treated with discontinued rapamycin and *Tsc1/Pkd1-BAC* transgenics. (**a**) H&E staining of kidney sections from controls (n=3), *$Tsc1^{f/f}$:KspCre* (n=9) and *$Tsc1^{f/f}$:Pkd1*-BAC:*KspCre* (n=9) mice at P30. Scale bar, 1000µm. (**b**) Left. H&E staining of kidney sections from P50 control and *$Tsc1^{f/f}$*:KspCre (n=3) mice. Middle. H&E staining of P50 *$Tsc1^{f/f}$:KspCre* mice (n=3) treated (Treatment I) with rapamycin 1mg/kg given to the feeding mother from P3 to P10. From P10 rapamycin was administered 1mg/kg directly to pups until P20. Right. H&E staining of P50 *$Tsc1^{f/f}$*:KspCre (n=2) mice treated (Treatment II) with rapamycin 1mg/kg from P8 to P20. Rapamycin treatment was interrupted from P20 to P50 when kidneys were collected. Scale bar, 1000µm.

Figure 1c

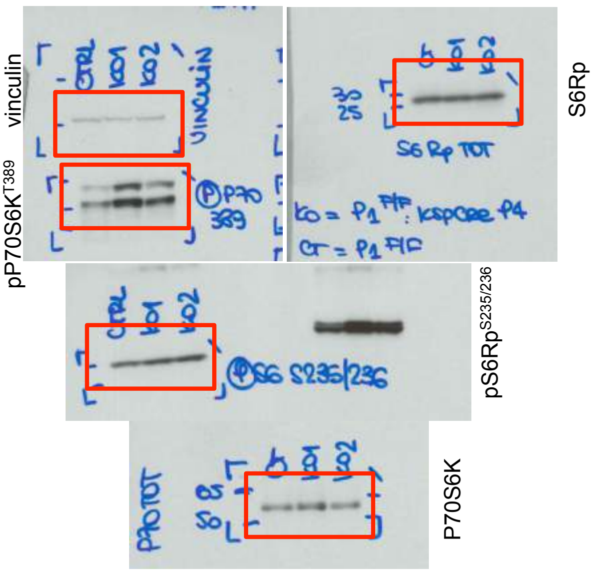

Figure 1d

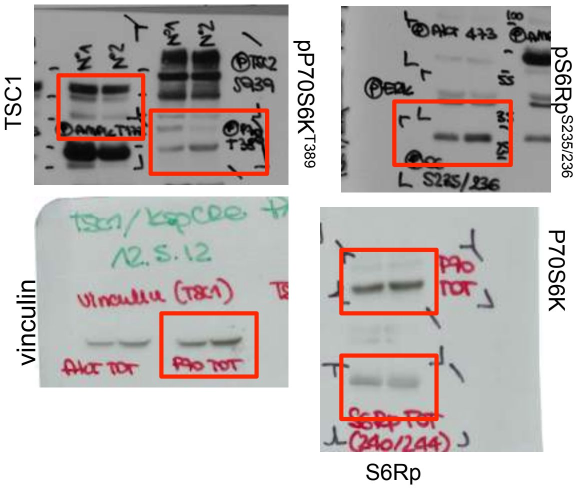

Figure 2a

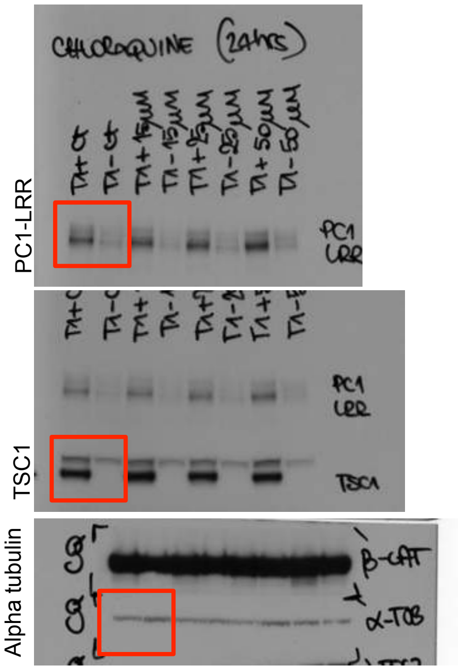

Figure 2b

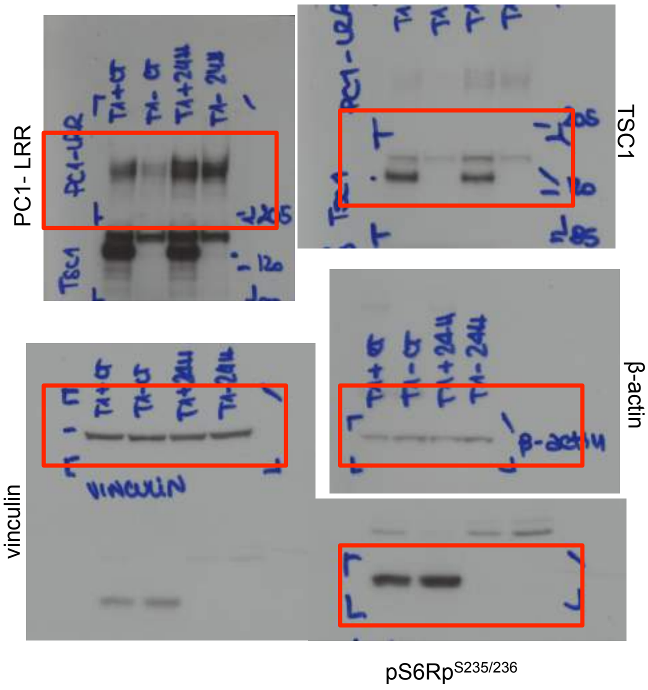

Supplementary Figure 7. Full blots for the western blots shown within the paper

Figure 2c

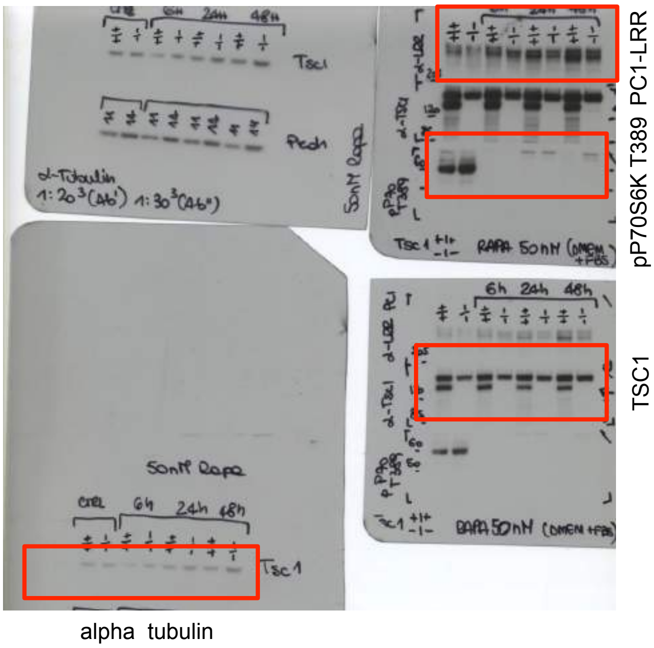

Figure 2d

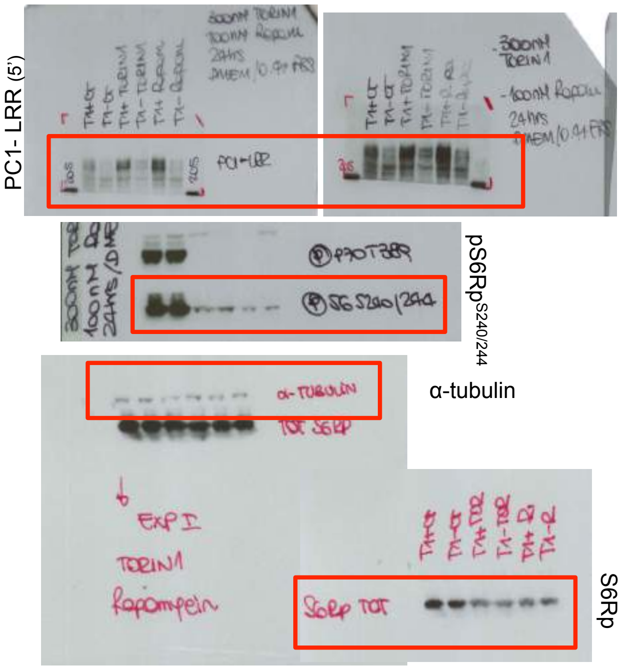

Figure 2e

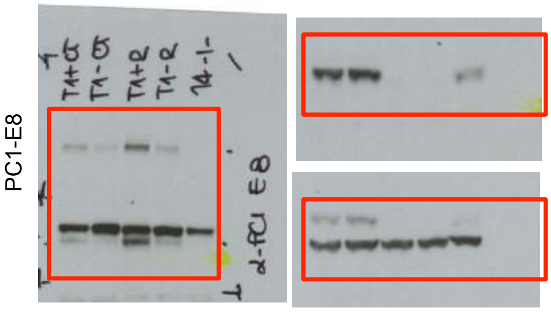

Figure 2f

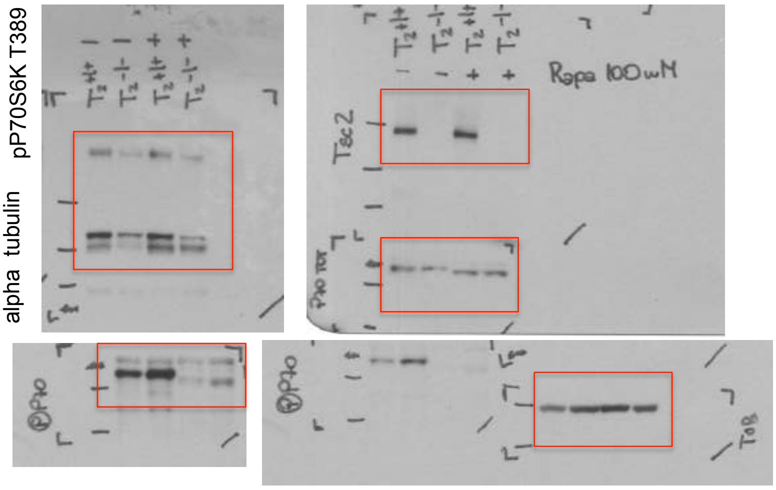

Figure 2g

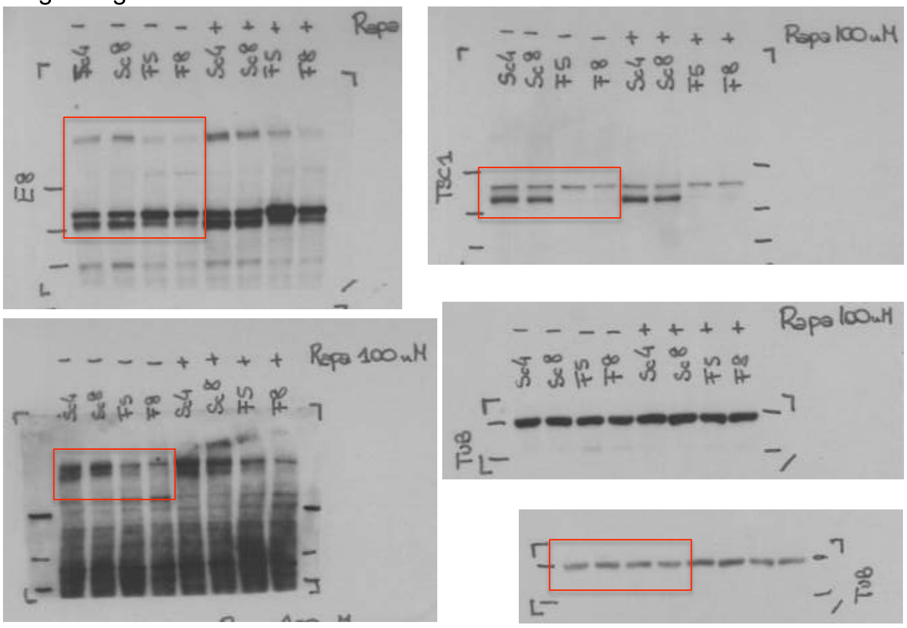

Supplementary Figure 7. Full blots for the western blots shown within the paper

Figure 3a

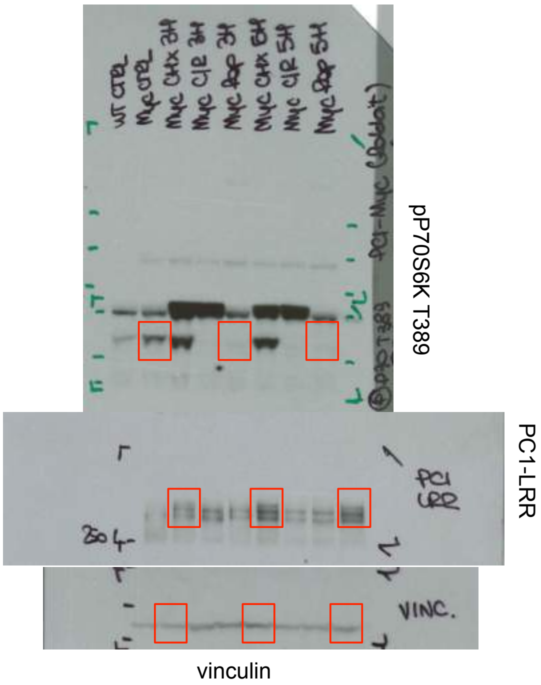

Figure 3c

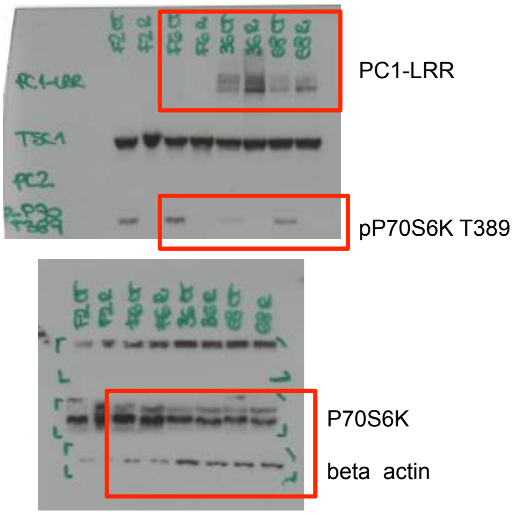

Figure 3d

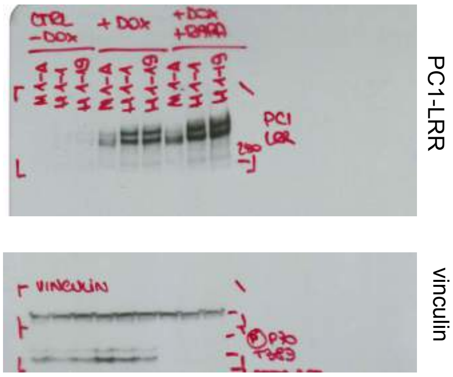

Figure 3e

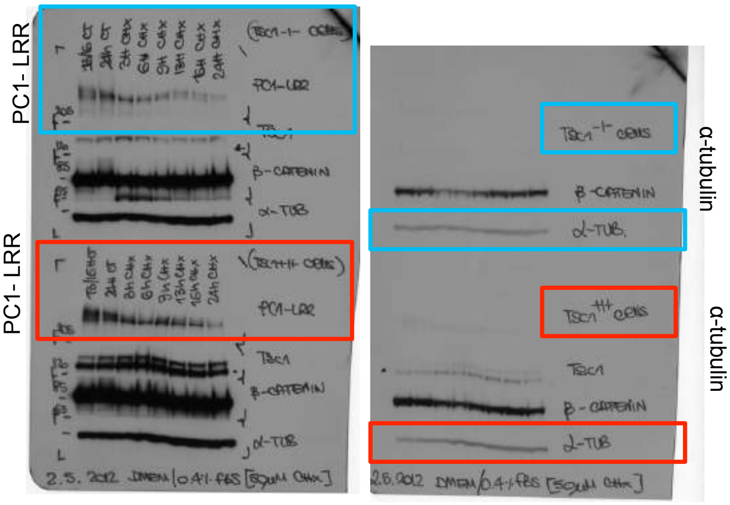

Figure 3g

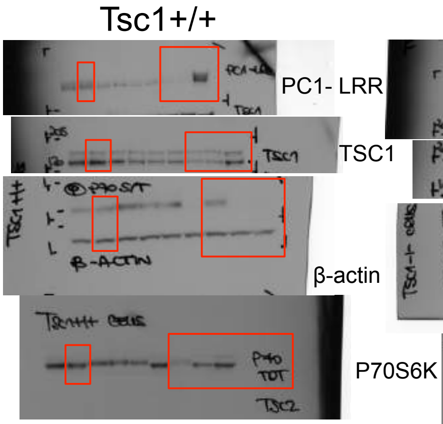

Figure 3h

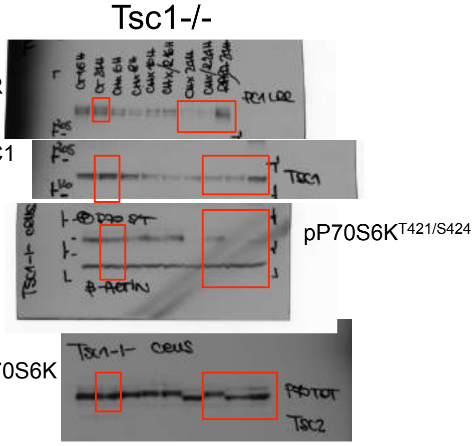

Figure4a

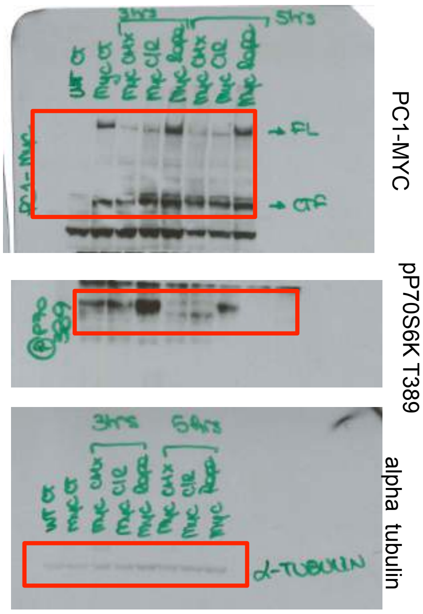

Figure 4c

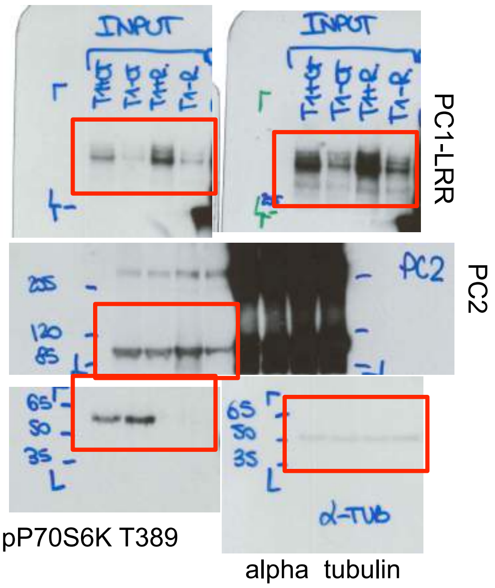

Figure5l

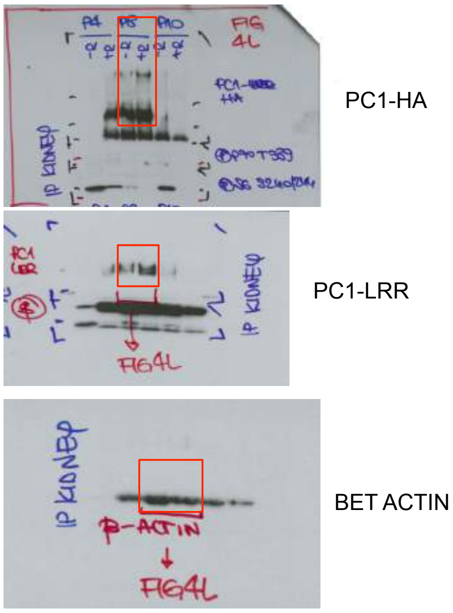

Supplementary Figure 7. Full blots for the western blots shown within the paper

Figure S1a

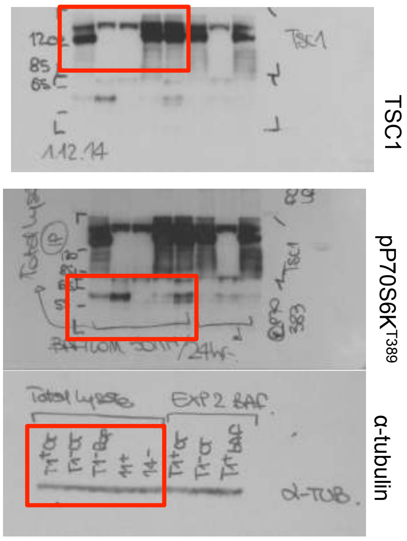

Figure S1b

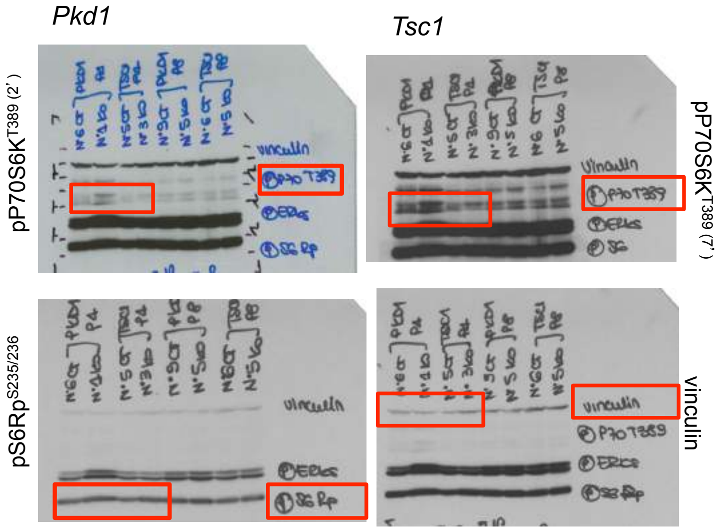

Supplementary Figure 1d_left

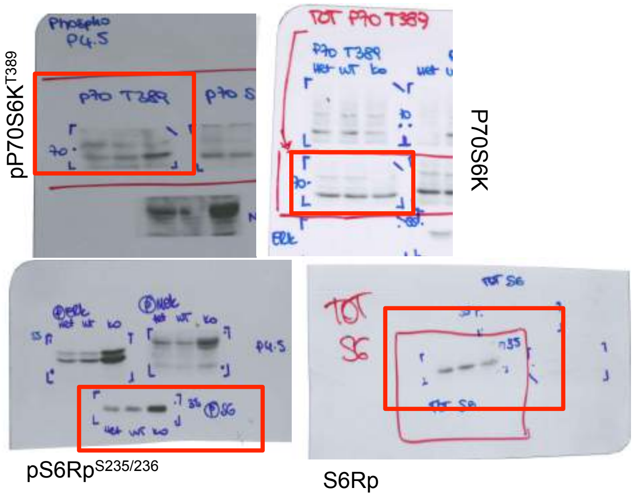

Supplementary Figure 1d_right

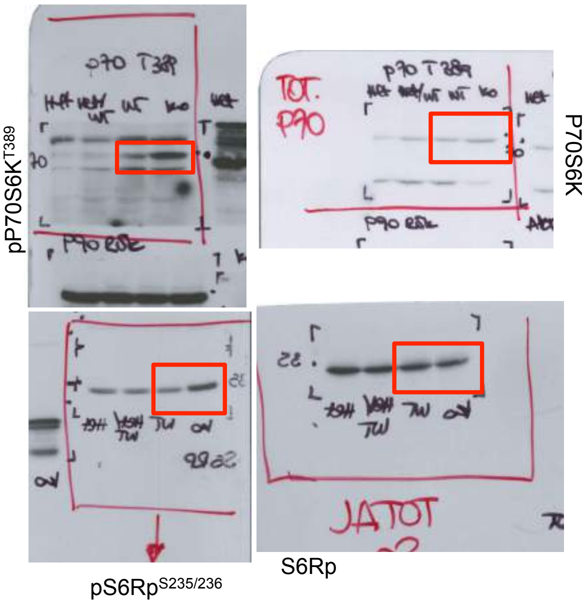

Supplementary Figure 1f

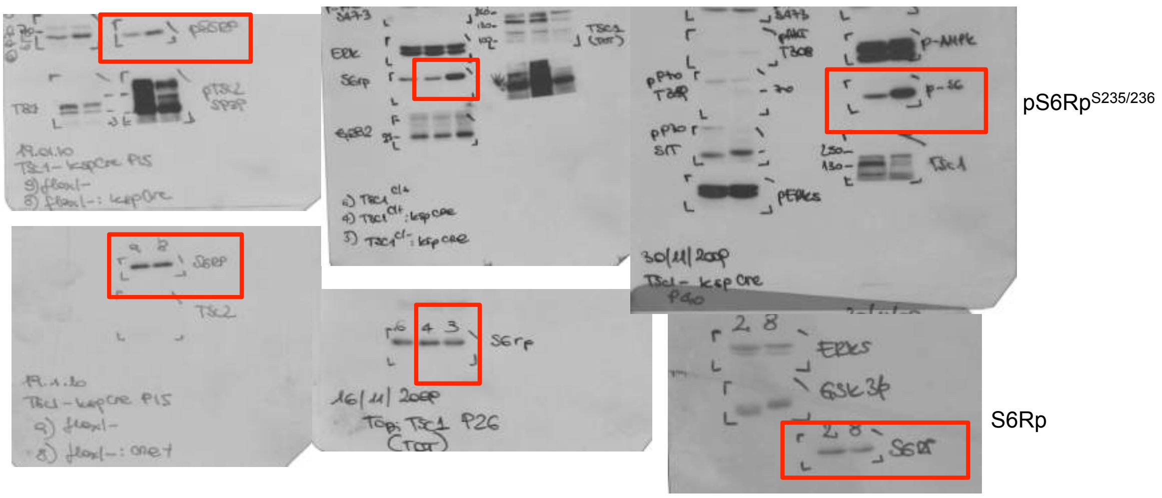

Supplementary Figure 7. Full blots for the western blots shown within the paper

Supplementary Figure 2a

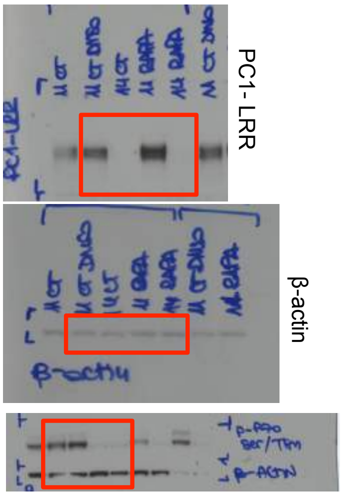

Supplementary Figure 2b

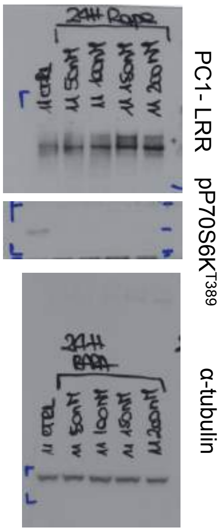

Supplementary Figure 2c

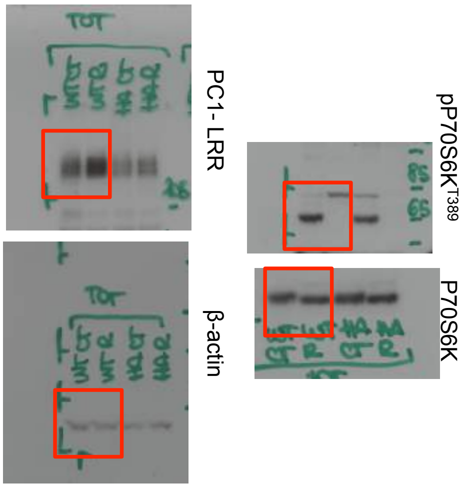

Supplementary Figure 2d

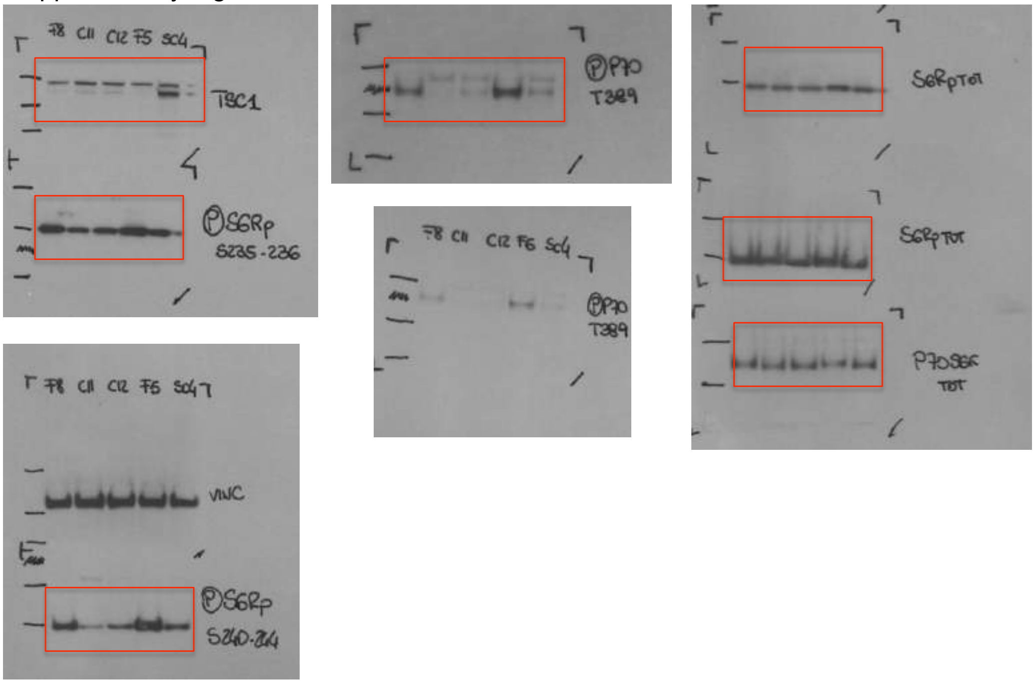

Supplementary Figure 7. Full blots for the western blots shown within the paper

Supplementary Figure 3d

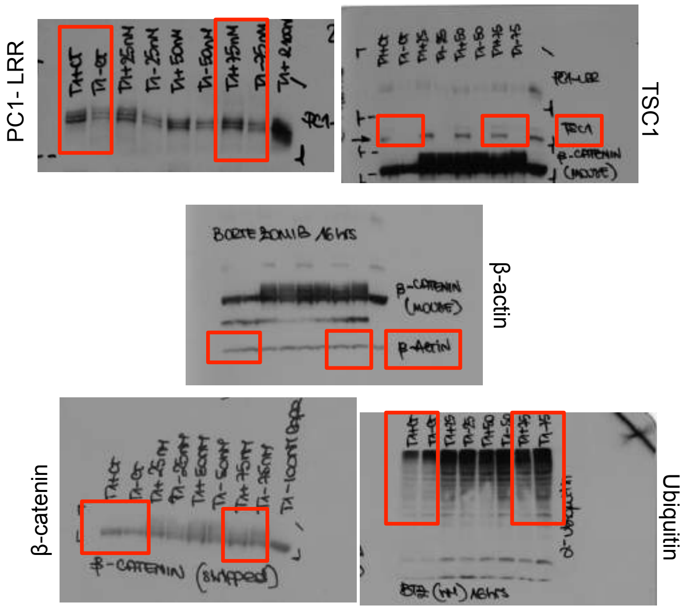

Supplementary Figure 3e

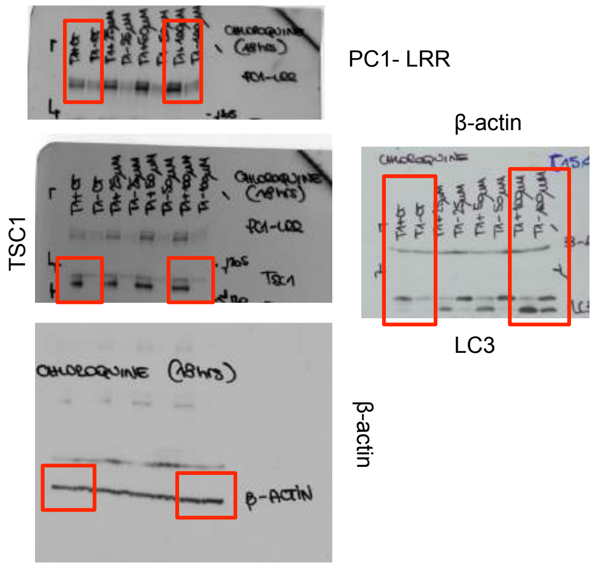

Supplementary Figure 3f

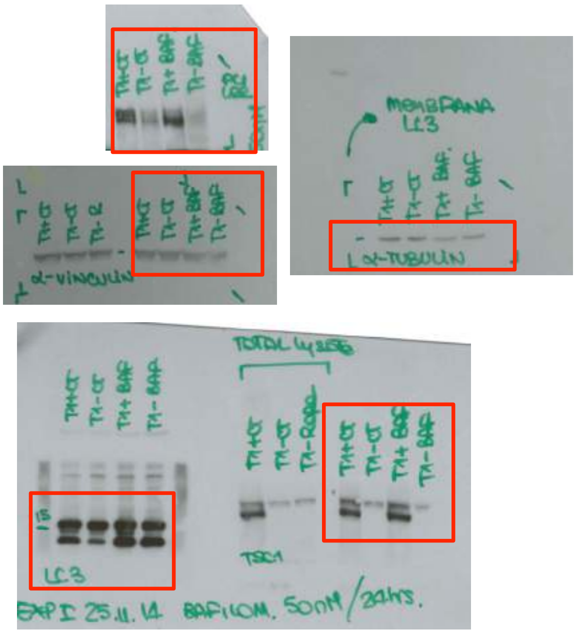

Supplementary Figure 4a

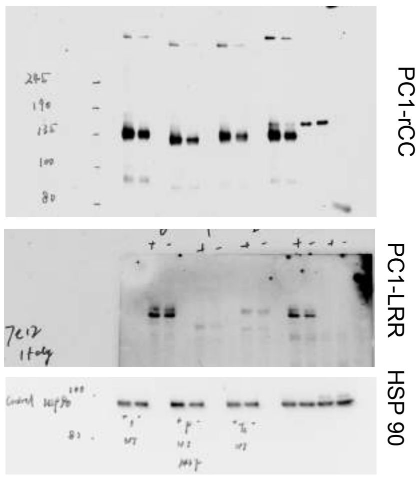

Supplementary Figure 7. Full blots for the western blots shown within the paper
